# Autophagic reprogramming of bone marrow–derived macrophages

**DOI:** 10.1007/s12026-022-09344-2

**Published:** 2022-12-01

**Authors:** Mayada Mazher, Yomna Adel Moqidem, Mona Zidan, Ahmed A. Sayed, Ahmed Abdellatif

**Affiliations:** 1 Biotechnology Program, School of Sciences and Engineering, The American University in Cairo, 11835 Cairo, Egypt; 2 Children’s Cancer Hospital, 57357 Cairo, Egypt; 3 Department of Biology, School of Sciences and Engineering, The American University in Cairo, Cairo, 11835 Egypt

**Keywords:** Macrophages, Autophagy, Macrophage polarization, Autophagy-related genes, M1 macrophages, M2 macrophages

## Abstract

Macro-autophagy is a highly conserved catabolic process among eukaryotes affecting macrophages. This work studies the genetic regulatory network involving the interplay between autophagy and macrophage polarization (activation). Autophagy-related genes (Atgs) and differentially expressed genes (DEGs) of macrophage polarization (M1–M2) were predicted, and their regulatory networks constructed. Naïve (M0) mouse bone marrow–derived monocytes were differentiated into M1 and M2a. Validation of the targets of Smad1, LC3A and LC3B, Atg16L1, Atg7, IL-6, CD68, Arg-1, and Vamp7 was performed in vitro. Immunophenotyping by flow cytometry revealed three macrophage phenotypes: M0 (IL-6 + /CD68 +), M1 (IL-6 + /CD68 + /Arg-1 +), and M2a (CD68 + /Arg-1). Confocal microscopy revealed increased autophagy in both M1 and M2a and a significant increase in the pre-autophagosomes size and number. Bafilomycin A increased the expression of CD68 and Arg-1 in all cell lineages. In conclusion, our approach predicted the protein targets mediating the interplay between autophagy and macrophage polarization. We suggest that autophagy reprograms macrophage polarization via CD68, arginase 1, Atg16L1-1, and Atg16L1-3. The current findings provide a foundation for the future use of macrophages in immunotherapy of different autoimmune disorders.

**Supplementary Information:**

The online version contains supplementary material available at 10.1007/s12026-022-09344-2.

## Introduction

Macrophages are major players in the immune system, and their phagocytic function contributes to host–pathogen defense mechanisms [1]. The activation of macrophages affects the quality of phagocytosis [2]. Autophagy is a highly conserved cellular catabolic process essential for cellular recycling that regulates phagocytosis in macrophages through modulation of the surface receptors [3–7]. Autophagy is also involved in the pathophysiology of many diseases such as neurodegenerative disorders [8], tumorigenesis [9], diabetes [10], and the immune response to infections [11]. The process starts with phagophore formation and elongation, autophagosome formation, and lysosomal fusion, followed by autolysosomal degradation [12].

Autophagy is initiated in response to starvation or amino acid depletion via inhibiting the nutrient-sensing kinase mammalian target of rapamycin complex 1 (mTORC1) and activating the AMP-activated protein kinase (AMPK) pathway, which activates mammalian Atg1/ULK1 kinase [13, 14]. Atg16L1 is a component of the phagophore elongation complex (Atg5–Atg12–Atg16L1) [15–19]. Atg16L1 and Atg9 regulate autophagosome formation by enhancing the conjugation of phosphatidylethanolamine (PE) with 1A/1B light chain 3 (LC3) (Atg8-like) to form LC3-II (MAP1LC3A, MAP1LC3B, and MAP1LC3C) [20, 21]. LC3 is critical for autophagosome–lysosomal fusion [22].

Mature autophagosomes [23] fuse with lysosomes to degrade autophagosome contents. The lysosomal protein vesicle-associated membrane protein 7 (Vamp7) is essential for the phagocytosis of opsonized particles [24–26].

Autophagy regulates the secretion of pro-inflammatory cytokines, such as IL-1β [27], IL-23 [28], IL-18, interleukin 6 (IL-6), and IL-1α [29]. Interferon gamma (INF-γ) induces autophagy via increasing the autophagosome formation and the turnover of LC3-II protein through the interferon regulatory factor 1 (IFR-1) signaling pathway [30], and it also mediates the upregulation of STAT1 and STAT2 in human peripheral blood mononuclear cells and macrophages [31].

According to their polarization state, inflammatory macrophages are classified into pro-inflammatory M1 macrophages and anti-inflammatory M2 macrophages [32–34]. Previous studies reported in vitro polarization of macrophages with IFN-γ, lipopolysaccharide, and interleukin 4 (IL-4) or IL-13 and showed high levels of IL-6 in the M2 phase [35]. The inhibition of the IL-6/STAT3 pathway with anti-IL-6 treatment caused M2 to change into M1 type.

Here, we investigate how autophagy reprograms macrophage polarization, as the interplay between autophagy and macrophage polarization is poorly understood. Finding the targeted proteins that mediate the interplay between autophagy and macrophage polarization among a pool of autophagy-related proteins and hundreds of growth factors and proteins that regulate macrophage polarization is quite challenging. Therefore, we implemented a systems biology approach to narrow down the protein targets that mediate the interplay between the two processes. These target proteins were validated in vitro using bone marrow–isolated macrophages.

## Methods

### In vitro isolation and polarization of macrophages

#### Ethical disclosure

All procedures were performed in compliance with the National Institutes of Health (NIH) guidelines for the Care and Use of Laboratory Animals (NIH Publications No. 8023, revised 1978), and according to Directive 2010/63/EU of the European Parliament and of the Council of 22 September 2010 on the protection of animals used for scientific purposes. All methods are reported in accordance with ARRIVE guidelines.

##### Isolation and characterization of bone marrow–derived monocytes

Female C57B/6 J mice were euthanized by an overdose of ketamine xylazine followed by cervical dislocation. The femur and tibia were removed and rinsed in ethanol 70% for 5 min, followed by 1 × phosphate-buffered saline (PBS), 6.7 mM PO_4_, without calcium and magnesium. The tibia and femur were rinsed in Dulbecco’s modified Eagle’s medium: F12, DMEM:F12 with HEPES (25 mM), 1:1 mixture with 3.151 g/l glucose, with l-glutamine (Lonza, Basil, Switzerland) for 10 min. The bones were flushed with 1 × PBS over a 70-µm cell strainer (Greiner, Kremsmünster, Austria).

The cell suspension was lysed with 1 × ammonium–chloride–potassium lysing buffer saline (Lonza, Basil, Switzerland) for 5 min to eliminate red blood cell and thrombocyte contamination. Following the lysis, the cell suspension was centrifuged for 5 min at 500 g. The cells were resuspended in lymphocyte separation medium (Lonza Basil, Switzerland) combined with DMEM/F12 Complete Medium (DMEM/F12 + 10% FBS + 1% penicillin and streptomycin) and centrifuged at 500 g for 10 min. The cell suspension was collected, counted, and seeded at a density of 300,000 cells/well in 12-well plates (Greiner, Kremsmünster, Austria) and incubated for 72 h, at 37 °C and 5% CO_2_.

##### M1–M2a lineage polarization

Monocytes were maintained in complete DMEM/F12 medium (DMEM/F12 + 20% L929 conditioned medium + 10% FBS + 1% penicillin and streptomycin). Mouse skin fibroblast cell line L929 was used as a source for monocyte colony-stimulating factor (M-CSF) for alternative activation of bone marrow–derived macrophages as previously reported [50]. Five days after isolation, naive macrophage lineage (M0) was polarized to M1 using type II interferon gamma (1250 IU/ml; STEMCELL Technologies, Cambridge Research Park, UK). M2a was polarized using interleukin-4 (2500 IU/ml; Cambridge Research Park, United Kingdom) in combination with 10 ng/ml lipopolysaccharide (LPS) [51] from *Escherichia coli* (Thermo Fisher Scientific, Waltham, MA, USA) for 48 h as previously reported [52]. On day 7, cells were polarized to reach either M1 or M2a lineage for further experimental use.

##### Cell viability and cytotoxicity assay

Macrophages were seeded in 96-well plates (10,000 cells/well). MTT tetrazolium reduction assay was performed as previously reported [53]. In summary, following a 3-h incubation with MTT reagent, the media were removed, and DMSO was added to dissolve the formazan crystals. The cells were examined using an inverted microscope (Olympus 1X70, Tokyo, Japan), and absorbance was measured at 570 nm using a microplate reader (Ultrospec 3100 pro). Cell viability (%) was calculated based on the following equation:

$$\mathrm{Survival\; rate }(\mathrm{\%})=(\mathrm{Ab\; sample}-A\mathrm{b\; blank})/(\mathrm{Ab \;control}-\mathrm{Ab \;blank})\times 100$$

where Ab sample is the sample absorbance, Ab blank is the absorbance of blank, and Ab control is the absorbance of the control.

##### Autophagy assay

On day 5, naïve macrophages (M0) were seeded at 96-well plates at a seeding density of 10,000 cells/well for 48 h. Autophagy assay was performed according to the manufacturer’s instruction (MAK138 fluorometric assay kit; Sigma-Aldrich, Saint Louis, MO, USA). The media was removed, and autophagosome detection reagent was added and incubated in the dark for 1 h at 37 °C and 5% CO_2_. Cells were washed gently by adding 100 µl of washing buffer, and the fluorescence intensity was measured (*λ*_ex_ = 360/*λ*_em_ = 520 nm).

##### Phagocytosis assay

Naïve macrophages were seeded at day 5 into 96-well plates at a seeding density of 10,000 cells/well to contain a final volume 100 µl/well primed for 48 h to M1 and M2a lineages as previously mentioned. Cells were stained with MAK138 autophagosome detection reagent as mentioned earlier. *E. coli* top 10 bacteria were grown in LB broth liquid (purchased from Thermo Fisher Scientific, Waltham, MA, USA) and were added to the cells. Cells were stained with 1 µg/ml 4′,6-diamidino-2-phenylindole·2HCl (DAPI) stain (Lonza, Basil, Switzerland) and examined under fluorescent microscopy (inverted fluorescent microscope; Leica Microsystems, Germany). Phagocytic events were counted for each condition.

##### Early apoptosis detection

Macrophages were primed to M1 and M2a as previously described. SH-SY5Y neuroblastoma cells (ATCC CRL-2266) were cultured in conditioned media from naïve macrophages (M1 and M2a) for 24 h. Cells were fixed with 4% PFA and permeabilized for 10 min with 0.3% triton X-100. Cells were washed and stained with DAPI and mounted on slides. Cells were examined under the microscope (inverted fluorescent microscope; Leica Microsystems, Germany). Cells treated with 20 ng/ml cisplatin were used as a positive control.

##### Immunofluorescent staining

Macrophages were fixed with 4% PFA for 10 min and washed with PBS. Cells were blocked and permeabilized with blocking buffer (5% BSA, 0.3% Triton X-100 in 1 × PBS) for 1 h. Cells were incubated overnight at 4 °C in the dark with the following primary antibodies: rabbit Mab LC3B (1:200), rabbit Mab Atg16L1 (1:100), rabbit Mab Smad1 (1:200), rabbit Mab Atg7 (1:200), and rabbit Mab IL-6 (1:200) (Cell Signaling Technologies, Danvers, MA, USA). Cells were later incubated with anti-rabbit Mab polyclonal secondary antibody for 2 h (Alexa Flour 488, 1:500), followed by washing and DAPI counterstaining for 10 min. Cells were examined under a fluorescence microscope (fluorescent microscope; Leica Microsystems, Germany). For confocal microscopy, a Leica Microsystems laser confocal microscope was used. Images were deconvoluted using Carl Zeiss Zen Blue 12 (Carl Zeiss, USA) software, and Z-stacks were 3D reconstructed using ICY software [54].

To detect intracellular trafficking of Atg7, Atg16l1, and LC3B inside the cytoplasmic or nuclear compartment, an automated spot detector plug-in SICE was used as described by Bayle et al. [55]. Images were taken by a fluorescent microscope (Leica Microsystems, Germany) and imported to ImageJ® software. A minimum of 8 images was counted for each condition.

##### Flow cytometry

Macrophages were collected and washed with 0.5% FBS in 1 × PBS and centrifuged at 350 g for 5 min. Cells were stained with mouse-specific antibody conjugate eFlour660 CD68 and Alexa Flour 488 conjugated arginase 1 (eBioscience, USA) for 30 min and washed with 1 × PBS at 500 g for 10 min. Unstained samples were used as a negative control. Samples were measured and gated on a flow cytometer (CytoFLEX, Beckman Coulter, USA, using two lasers: red laser (with an excitation wavelength of 660 nm) for allophycocyanin (APC) and blue laser (with an excitation wavelength of 488 nm) for fluorescein isothiocyanate (FITC).

### Statistical analysis

Statistical analyses were carried out using GraphPad Prism® software. Data was expressed as mean ± standard deviation, or median and range were used for data expression. All tests were two-tailed. Post hoc tests and one-way ANOVA were used to compare the differences of mean values between different groups. *p* values that were less than 0.05 were considered statistically significant.

## Results

### In silico analysis of autophagy-related genes

We used a network-based systems biology approach to model the interplay between the complex signaling pathways of autophagy and macrophage polarization. The analysis of the different databases identified common significantly enriched pathways and common regulatory transcription factors that co-regulate both transcription factors and Atgs and M1–M2–DEGS (Supplementary Data).

### In vitro isolation and polarization of bone marrow macrophages

**Fig. 1:**
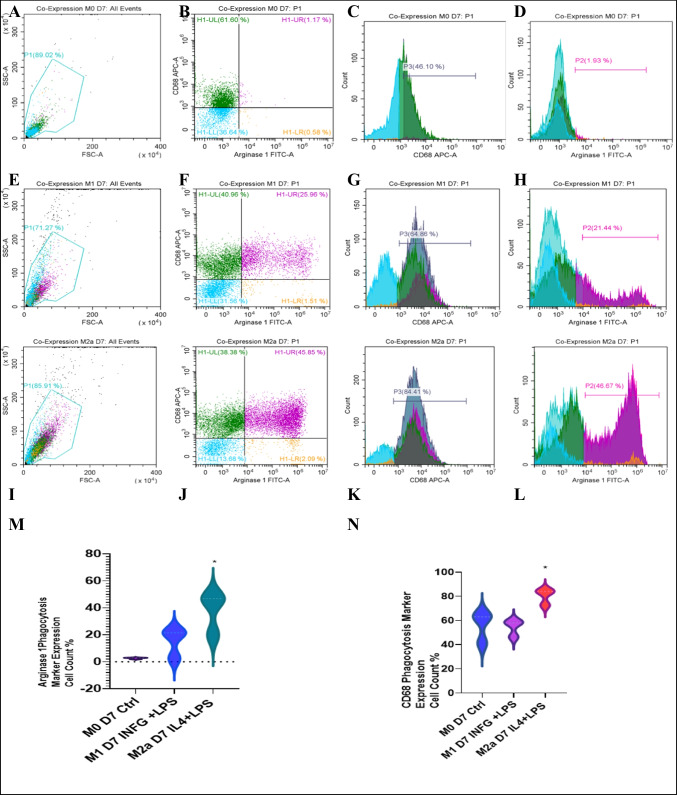
Co-expression of CD68 and arginase 1 in macrophages at day 7. Flow cytometry analysis for M1 and M2a, using M0 macrophages as a control. Samples were gated on 81%, CD68 expression was assessed using an APC filter, and arginase 1 was read using a FITC filter. **A**, **E**, and **I** show the gated cells (M0, M1, and M2a lineages), respectively, on FSC-H and SSC-H. **B**, **F**, and **J** are quadrant plots for M0, M1, and M2a, respectively. **C**, **G**, and **K** are histogram fluorescence peak signal plots for CD68 expression in M0, M1, and M2a cells. **D**, **H**, and **L** are fluorescence peak signal plots for arginase 1 expression in M0, M1, and M2a. Statistical analysis for the expression of arginase 1 (**M**) and CD68 (**N**) in bone marrow–derived macrophages at day 7 showed that M2a lineage significantly expressed both CD68 and arginase 1 compared to M0 and M1 (*n* = 3, *p value =  < 0.5). No expression of arginase 1 in M0 lineage was seen

**Fig. 2:**
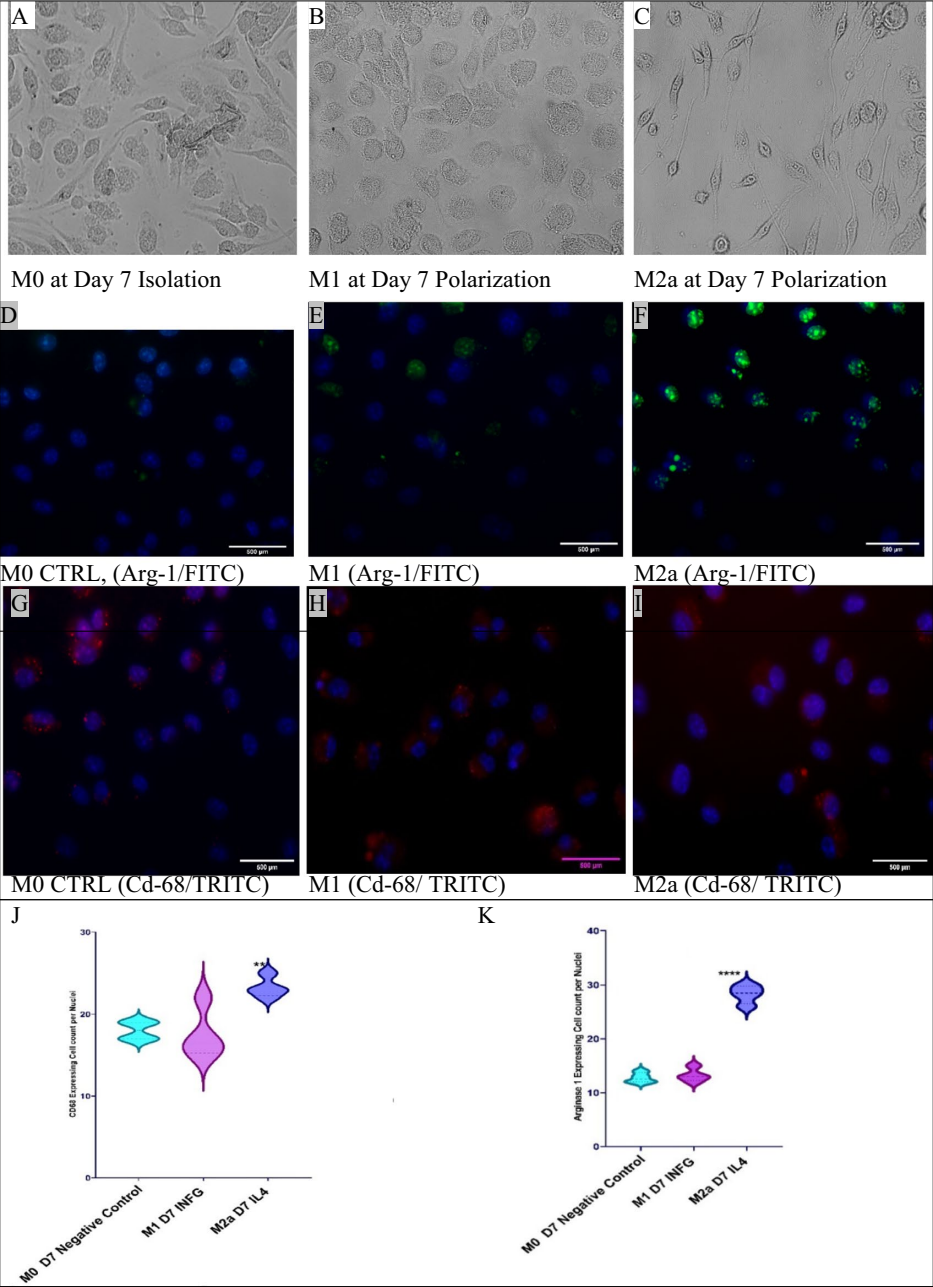
Microscopic examination of bone marrow–derived macrophages. Morphological examination for bone marrow–derived macrophages. **A** represents the fully differentiated M0 by using L929 conditioned medium 20 ng/ml at day 7. **B** represents the fully differentiated M1 activated by INF-γ (1250 IU/ml) + LPS (100 ng/ml) for (48 h) at 7-day polarization. **C** represents the fully differentiated M2a activated by IL-4 (2500 IU/ml) + LPS (100 ng/ml) for (48 h) at 7-day polarization. **D**, **E**, and **F** show the expression of both phagocytosis markers CD68 (cell surface and intracellular) and Arg-1 (intracellular). M0, M1, and M2a cells (**D**, **E**, and **F**, respectively) stained with arginase 1 read using a FITC filter and counterstained with DAPI. The expression of intracellular arginase 1 can be seen in green. **G**, **H**, and **I** are stained with CD68 red (TRITC) and counterstained with DAPI. Manual cell counting was performed for cells expressing CD68 (**J**) and Arg-1 (**K**) in M0, M1, and M2a lineages on ImageJ.® using the cell counter plugin (*n* = 4, **p value = < 0.01, ****p-value = <0.0001 )

Murine bone marrow monocytes were isolated and differentiated to M0 using 20% L929 conditioned media. On day 5, type II interferon-γ was used (1250 IU/ml) combined with LPS (100 ng/ml) for 48 h to activate the M0 into M1 lineage. For M2a, IL-4 was used (2500 IU/ml) in combination with LPS (100 ng/ml) for 48 h. The three lineages were characterized using flow cytometry with three markers: interleukin-6, CD68, and arginase 1 (Fig. 1, Supp. Data Fig. 2S).

The phenotypes of the isolated cells were verified using CD68 and arginase 1. CD68 was expressed in all cell phenotypes, although M2a showed a significantly higher expression of 84%. Flow cytometry analysis showed a significant increase in total expression of arginase 1 in M2a phenotype more than M1 and was absent in the control M0 lineage. The resulting phenotypes were M0 (IL-6 + /CD68 +), M1 (IL-6 + /CD68 + /Arg-1 +), and M2a (CD68 + /Arg-1 +). Flow cytometry showed that M2a lineage significantly expressed both CD68 (*n* = 3, *p* value = < 0.5 and *R*^2^ = 0.7) and arginase 1 (*n* = 3, *p* value = < 0.05) (Fig. 1 and Supp. Data Fig. 11S). Immunostaining showed that M0, M1, and M2a expressed arginase 1 (*n* = 4, *p* value =  < 0.0001, *R*^2^ = 0.9) and CD68 (*n* = 4, *p* value = < 0.05, *R*^2^ = 0.68) (Fig. 2).

#### Interferon-γ promotes IL-6 expression in M1 lineage, while IL-4 inhibits the IL-6 expression in M2a lineage

**Fig. 3:**
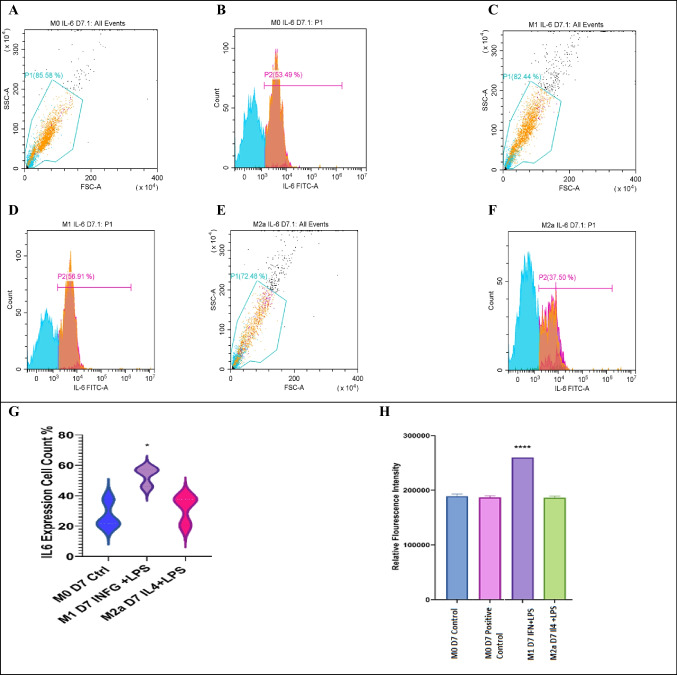
Expression of IL-6 by the flow cytometry. IL-6 expression by flow cytometry analysis in M1 and M2a, with M0 macrophages used as control. Samples were gated on 81%, and IL-6 expression was read using a FITC filter. **A**, **C**, and **E** represent the gating for 5000 events (event = single cell). **B**, **D**, and **F** are fluorescence peak signals for IL-6 percentage expression in M0, M1, and M2a cells. High expression of IL-6 in M0 and M1 was seen with very low expression in M2a macrophages. **H** shows a violin plot showing statistical significance for IL-6 expression (*n* = 3, *p* value = < 0.05). **I** shows a bar plot showing the expression of IL-6 protein terms of relative fluorescence intensity using a multi-plate reader. M0 (day 7) was used as control, and M0 + Earle’s balanced salt was used as positive autophagy control. M1 lineage significantly expressed IL-6 (*n* = 3, *p value = < 0.05, ****p value =  < 0.0001, *R*.^2^ = 1)

Flow cytometry studies show that interferon-γ stimulated M1 lineage expressing the phagocytosis marker IL-6 significantly (56%) compared to M2a lineage (37%) (Fig. 3). However, IL-6 expression was also high in the control M0 lineage. Also, the fluorescence intensity for IL-6 protein showed that M1 lineage had the most significant increase in IL-6 protein expression. Surprisingly, the conditioned media of M2a 7-day macrophages showed cytotoxic activity on neuroblastoma cell line SH-SY5Y (Supp. Data Fig. 11S).

#### Increased Atg16L1 expression in M1 and M2a lineages

**Fig. 4:**
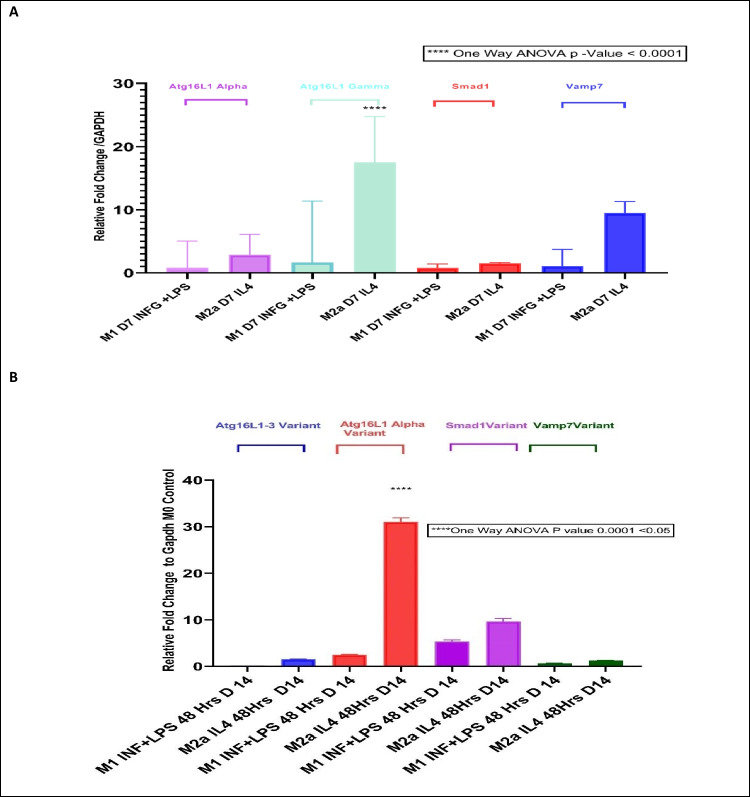
Summary of gene expression data at 7-day and 14-day polarizations. Summary of gene expression data at day 7 (**A**) and day 14 (**B**). A significance of fold increase in Atg16L1-3 and Vamp7 gene expression was seen in M2a lineage at day 7. An increase in Atg16L1-1 (but not Atg16L1-3) and Smad1 was seen in M2a lineage at day 14. These results indicate a high autophagic activity in the M2a lineage

Atg16L1 serves as a precursor for the homotypic fusion of lysosomal Vamp7/SNARE proteins for the pre-autophagosome formation and LC3 autophagosome maturation. Therefore, we examined the expression of the Vamp7 gene at M0, M1, and M2a lineages at 7-day and 14-day polarizations. Finally, M2a lineage showed the highest and the most significant Atg16L1-1, Atg16L1-3, and Vamp7 fold change in both 7-day and 14-day polarizations (Fig. 4).

Interestingly, Atg16L1-1 alpha showed a 30-fold increase in M2a day-14 polarization than in M2a 7-day polarization. Also, the Atg16L1-3 gamma variant showed an 18-fold increase in M2a 7-day polarization than in M2a 14-day polarization. The same as for Vamp7 in M2a 7-day polarization showed a tenfold increase than in M2a 14-day polarization (****p-value = < 0.0001) (Fig. 13S).

#### Atg16L1-1 and Atg16L1-3 are upregulated in M2a lineage

**Fig. 5:**
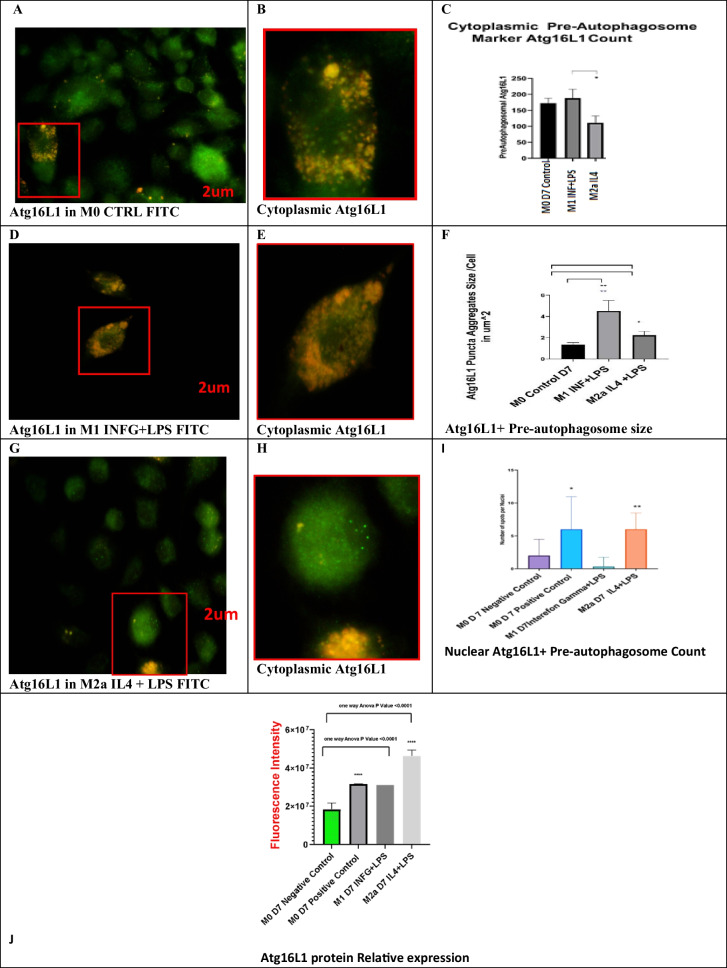
Immune co-localization of Atg16L1 + pre-autophagosomes. **A**, **B**, **D**, **G**, and **H** show the M0, M1, and M2a macrophages, respectively. Cytoplasmic pre-autophagosomes appeared as yellow to green spots. **C** shows the count of cytoplasmic pre-autophagosomes per cell (*n* = 6, *p* value = 0.027– < 0.05). **F** shows the size of pre-autophagosomes in µm.^2^ per cell (*n* = 6, *p* value = 0.0045). **I** shows nuclear Atg16L1 pre-autophagosome count (*n* = 3, *p* value = 0.036). M1 lineage showed the highest number and size of cytoplasmic pre-autophagosomes in the cytoplasm. **J** shows relative fluorescence intensity was measured using a multi-plate reader. M2a showed the most significant increase in fluorescence intensity (*n* = 3, *p value = <0.05, **p value = <0.01, ****p value =  < 0.0001)

M2a cell lineage showed upregulation for both Atg16L1-1 and Atg16L1-3 variants (Fig. 5). We were able to detect the pre-autophagosomes and their size in M0, M1, and M2a lineages. Cytoplasmic and nuclear pre-autophagosomes were stained for Atg16L1 (yellow to green spots, Fig. 5). M1 lineage showed the highest number of pre-autophagosomes in the cytoplasm (*n* = 6 images, at least 5 cells/image, *p* value = 0.0045– < 0.05) (Fig. 5). M1 was highly significant. However, M2a lineage showed a significant increase in cytoplasmic Atg16L1 spot size compared to M0 lineage control (*n* = 6 images, 5 cells/image, *p* value = 0.043– < 0.05, Fig. 4). Interestingly, the average size of Atg16L1 in M1 lineage is more than 4 µm diameter, which is above normal value for pre-autophagosome (from 500 to 1000 nm, 0.5–1 µm) diameter.

Immunolocalization using Atg16L1 in M0, M1, M2a, and M0 + Earle’s balanced salt revealed that no autophagosomes were observed in the M0 control and in M1 macrophages. However, we observed pre-autophagosomes in M2a lineage and in the positive autophagy M0 cells treated with EBS. M2a lineage showed a significant increase in both nuclear pre-autophagosome numbers (*p* value =  < 0.05) and cytoplasmic Atg16L1 size. This supports our gene expression data that showed increased fold change of Atg16L1-1 gene variation M2a at 7-day polarization (Fig. 5).

#### INF-γ increased Atg7 expression in M1 cells and increased pre-autophagosome size

**Fig. 6:**
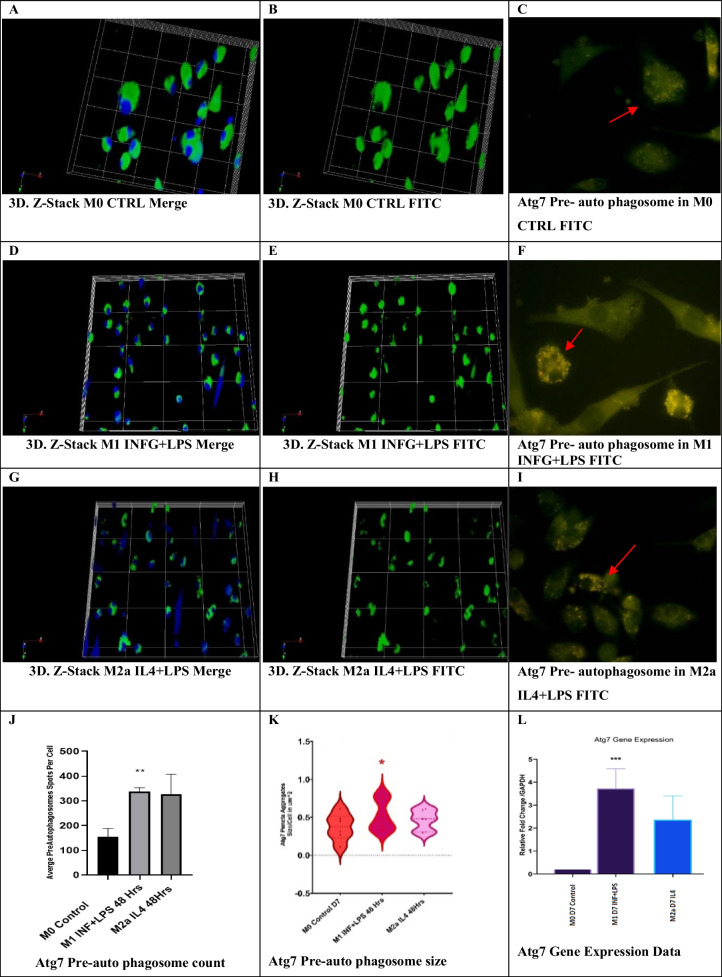
Immune co-localization of Atg7 using confocal microscopy. Three-dimensional reconstruction of Z-stack confocal microscopy images revealed a significant number of pre-autophagosomes formed at M1 and M2a lineages. M0 control showed the largest size of pre-autophagosomes. Atg7 expression is seen as green or yellow dots (pre-autophagosomes) distributed in the cytoplasmic compartment (red arrows). **J** shows the count of Atg7 + pre-autophagosomes per cell (*n* = 6, *p* value = < 0.05). No significant difference between M1 and M2a was seen. **K** shows a violin plot of Atg7 pre-autophagosome size in µm^2^ measured using ImageJ.®. M1 showed an increased spot size (*n* = 6, *p* value = < 0.05). **L** shows Atg7 gene expression was significantly upregulated in M1 cells (*n* = 4, *p value = < 0.05, **p value = <0.01, *** *p* value = < 0.001)

Immune co-localization studies show Atg7 expression as pre-autophagosomes distributed in the cytoplasmic compartment in M1 and M2a lineages. Confocal images revealed a significant number of pre-autophagosomes formed in M1 and M2a lineages. However, M0 control showed the largest size of pre-autophagosomes (Fig. 6). Statistical analysis of Atg7 and the pre-autophagosome number per cell show that there was no significant difference between M1 and M2a (300 spots/cell). M1 cells showed an increased pre-autophagosome size to more than 1 µm in diameter (*n* = 6 images, *p* value = < 0.05) (Fig. 6).

Relative fold change of gene expression normalized to GAPDH as endogenous control shows that the fold changes relative to GAPDH in M0 were as follows: mean = 3.7 and 2.36 folds, ± 0.46 and ± 0.56 for M1 and M2a, respectively (Fig. 6).

Therefore, INF-γ promoted the expression of the Atg7 protein and mediated upregulation of Atg7 gene expression in M1 and M2a cells. In contrast, INF-γ and lipopolysaccharide increased Atg7 protein and messenger RNA (mRNA) in M1 lineage.

#### Autophagy-associated protein complex LC3A and LC3B expression increased in M1 and M2a macrophages

**Fig. 7:**
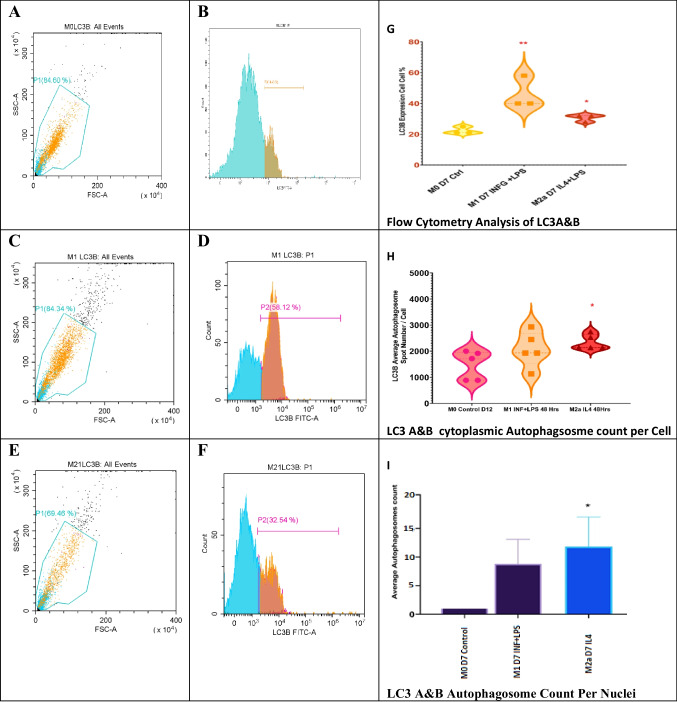
Flow cytometry analysis of LC3A and LC3B protein expression in bone marrow–derived macrophages. Samples were gated on 81%, and LCA and LCB protein expression was read using a FITC filter. **A**, **C**, and **E** represent the gating for 5000 events inside scatter plots for M0, M1, and M2a lineages, respectively. **B**, **D**, and **F** show fluorescence peak signals for LC3A and LC3B protein expression in M0, M1, and M2a cells, respectively. Higher expression of the LC3A and LC3B was seen in M1 and M2a, compared to M0. **G** shows a violin plot for total cells expressing LC3A and LC3B (*n* = 3, *p* value 0.01). **H** shows cytoplasmic LC3A and LC3B per cell (*n* = 5, *p* value = < 0.05). Manual counting of nuclear autophagosomes (**I**) showed that M2a was significantly higher (*n* = 5, *p value = < 0.05, **p value = < 0.01)

The MAP1-LC3s or LC3A and LC3B quantification showed that the distribution of autophagosomes inside the nuclear and cytoplasmic compartments is not uniformly distributed. However, autophagosomes were not localized in the nucleus in M1 and M0 control. The average number of basal autophagosomes in M0 was 1800. In M1, it was 2436 spot and in M2a increased to 2471 (Fig. 12S). Remarkably autophagosome aggregations were also observed. Flow cytometry single-cell quantification showed a significant increase in M1 and M2a cells (*p* value = 0.01– < 0.05, Fig. 7).

Finally, mRNA levels of LC3B but not LC3A increased in M1 to 4 folds and in M2a to 3 folds, respectively. Collectively, INFG + LPS induced macro-autophagy inside M1 and IL-4 + LPS induced macro-autophagy in M2a cells.

**Fig. 8:**
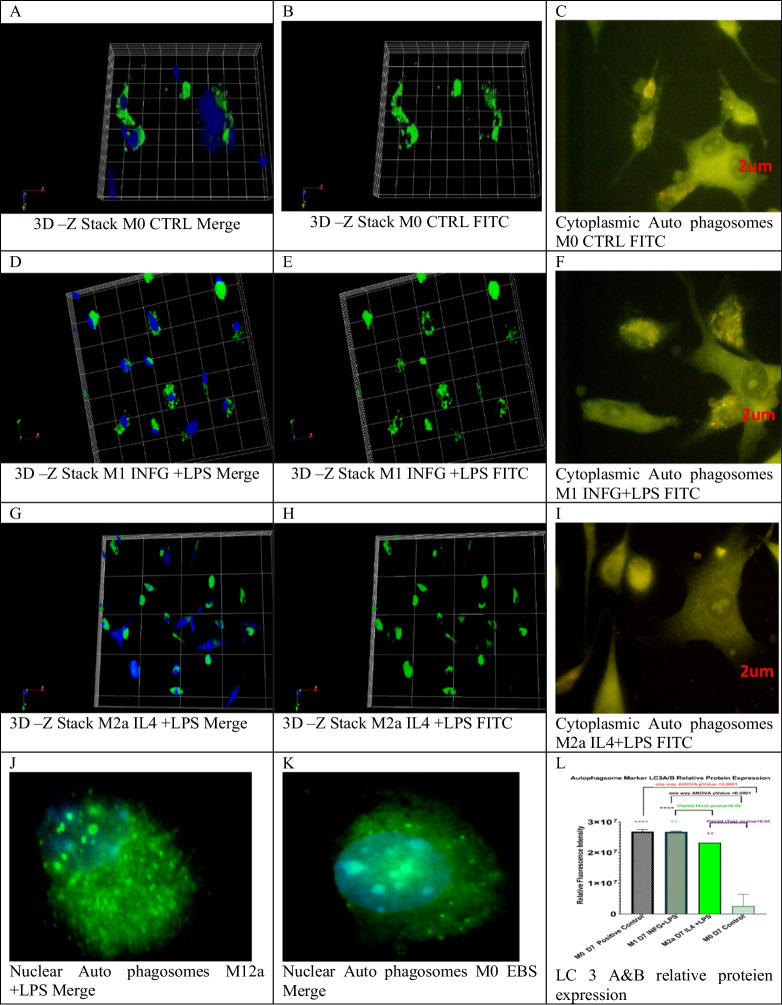
Immune co-localization of LC3A and LC3B protein complex using laser confocal microscopy. Reconstruction for Z-stack images revealed a significant number of autophagosomes formed at M0 and M1 lineages. M2a showed a large size of pre-autophagosomes (**B**, **E**, and **H**). **C**, **F**, and **I** show immune co-localization of cytoplasmic autophagosome (yellow to green spots) in M0, M1, and M2a lineages. LC3A and LC3B nuclear autophagosomes (yellow dots inside the nuclear compartment) (**J**, **K**). The number of autophagosomes was counted (**L**) inside the cytoplasm and in the nuclear using ImageJ®

High autophagosome aggregates were formed in both M1 and M2a lineages compared to M0 control (Fig. 8). Relative fluorescence intensity of LC3A and LC3B showed a significant increase in M1 and M2a lineages compared to M0 control (Supp. Data Fig. 12S).

#### Increased Smad1 gene expression in M1 and M2a lineages

**Fig. 9:**
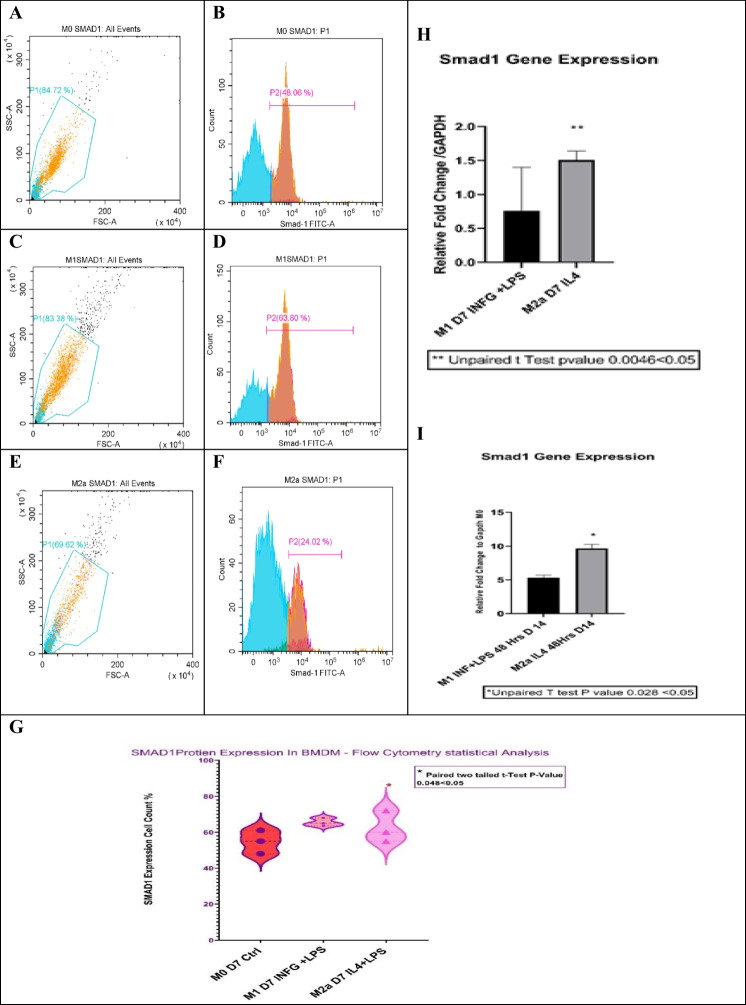
Flow cytometry analysis for Smad1 expression in bone marrow–derived macrophages. Smad1 flow cytometry analysis in M1 and M2a lineages using M0 macrophages as a control. Samples were gated on 81%, and Smad1 expression was read using a FITC filter. **A**, **C**, and **E** represent the gating for 5000 events (event = single cell) in scatter plots (SSC-A) on the *X*-axis and forwarded scatter plots (FSC-A) on the *Y*-axis. **A**, **C**, and **E** show M0, M1, and M2a lineages, respectively. **B**, **D**, and **F** show fluorescence peak signals for Smad1 expression in M0, M1, and M2a cells, indicating higher expression of Smad1 at M2a. **G** Violin plot shows statistical significance for Smad1 total expression (*n* = 3, *p value = < 0.05). Bar plot shows the statistical representation of Smad1 transcription factor fold change in both M1 and M2a at 7-day polarization (**H**) (*n* = 4, *p value = < 0.05) and 14-day polarization (**I**) (*n* = 4, *p value = < 0.05) normalized to GAPDH. M1 and M2a at 14-day polarization showed a significant increase in Smad1 gene expression

We report Smad1 as one of our predicted transcription factors and its downstream targets, IL-6, and MAPLC3A genes. Smad1 was downregulated in M1 and M2a at 7-day polarization compared with 14-day polarization results. Flow cytometry showed a significant difference in Smad1 protein expression at 7-day polarization in M2a compared to M0 and M1. Fold change in M2a cells at 7-day polarization was only 1.5 folds (*p* values =  < 0.05, Fig. 9). However, there was significant overexpression of Smad1 in both M1 and M2a lineages at 14-day polarization (*p* value =  < 0.05, Fig. 9).

#### Autophagy inhibitor bafilomycin A significantly increased CD68 and arginase 1 expression in M0, M1, and M2a Lineages

**Fig. 10:**
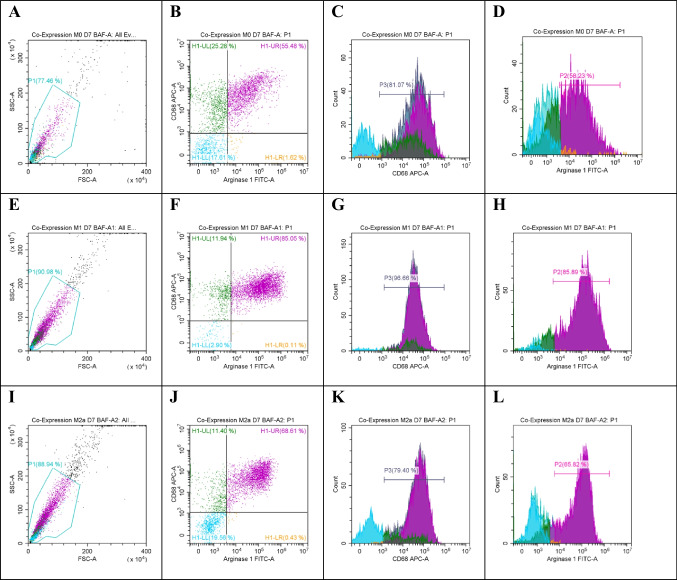
Co-expression of both CD68 and arginase 1 by flow cytometry analysis for M1 and M2a with bafilomycin A. Flow cytometry analysis for M0, M1, and M2a cells at 7-day polarization incubated with autophagy inhibitor bafilomycin A (200 nM). M0 macrophages were used as control. Samples were gated on 81%, and CD68 expression was assessed using an APC filter, and arginase-1 was read using a FITC filter. **A**, **E**, and **I** represent the gating for 5000 events for M0, M1, and M2a lineages, respectively. **B**, **F**, and **J** are quadrant plots for M0, M1, and M2a, respectively. **C**, **G**, and **K** are fluorescence peak signal plots for CD68 expression in M0, M1, and M2a cells. An increased expression of CD68 in all cell lineages was seen. **D**, **H**, and **L** represent the fluorescence signal peak for arginase 1 expression in M0, M1, and M2a. Autophagy inhibition (**D**) showed a great increase in arginase 1 expression (> 50%) in M0 lineage at 7-day polarization

Autophagy inhibition using bafilomycin A (200 nM) in M0, M1, and M2a cell lineages showed a high expression pattern for both arginase 1 and CD68 in M0, M1, and M2a cells. Also, bafilomycin A increased the percentage of double-positive (CD68/arginase1) cells in both M1 and M2a. Surprisingly, autophagy inhibition showed a great increase in arginase 1 expression more than 50% in M0 lineage at 7-day polarization compared to M0 at 7-day polarization with the normal basal autophagy activity (Figs. 10 and 14S).

#### Autophagy induction decreased the phagocytosis activity of M2a but not M1

The average number of phagocytic events in M2a lineage showed decreased phagocytic activity. However, no significant effect on M0 and M1 lineage was observed. Immune staining studies using Mak38 autophagy detection kit showed that autophagy decreased the phagocytic activity of M2a compared to M1 and M0 (Supp. Data Fig. 15S).

## Discussion

Autophagy depends on the formation of double-membrane autophagosomes that fuse with the lysosome to degrade pathogens, proteins, and organelles. Both phagocytosis and autophagy are interdependent processes. The interplay between autophagy, macrophage activation, and phagocytosis is still poorly understood [60, 61].

In this study, we dissected both the autophagy and macrophage activation process to understand the nature of this interplay. We were able to identify a list of common pathways, transcription factors, and target proteins that mediate this interplay (Supplementary Data). We further validated these targets in an in vitro study.

The predicted target proteins, Atg7, and Atg16L1 serve as central proteins for several signaling pathways in autophagy, macrophage polarization, and phagocytosis. Nevertheless, more experimental validation is needed for other predicted targets. Atg16L1 mediates the pre-autophagosome formation, which is essential for interaction with the Atg5–Atg12 complex that mediates the conjugation with PE [70].

Bone marrow–derived macrophages are a heterogeneous population. To characterize the phenotypes of the isolated bone marrow–derived macrophages and the activated macrophages in vitro, we investigated the expression of phagocytic markers CD68, IL-6, and arginase 1 among various macrophage populations. CD68 is a cell surface heavily glycosylated glycoprotein localized near the endosomal/lysosomes compartment, that is commonly used as a phagocytic marker in dendritic cells and strongly expressed in total macrophages, including M1 and M2 [78, 79]. It is also a marker of tumor-associated macrophages [80]. M0 and M1 macrophages were confirmed by the high expression of CD68 (more than 60%).

Arginase 1 is a novel marker for activated M2a cells [81]. In M1 cells, the Arg-1 + expression was 20%, and in M2a, Arg-1 + expression was 46%. However, M0 showed a rare expression for Arg-1 (less than 2%). Flow cytometry analysis and immunostaining studies showed strong expression of CD68 in both M1 and M2a, and the absence of arginase 1 in M0. Altogether, these data positively characterize all lineages, M0, M1, and M2a [82, 83].

IL-6 is a pro-inflammatory cytokine that we predicted to mediate the interplay between autophagy and macrophage activation. Interferon-γ and lipopolysaccharide combination promoted the expression of IL-6 in M1 lineage. Besides phagocytosis, cytotoxic activity is one of the characteristics of bone marrow–derived macrophages [84].

Flow cytometry studies on LC3A and LC3B protein expression revealed that interferon-γ and lipopolysaccharide induced macro-autophagy in IL-6 + /CD68 + M1. Also, Interleukin 4 and lipopolysaccharide combination induced macro-autophagy in Arg-1 + /CD68 + M2a macrophages.

In the current study, INF-γ and IL-4 in combination with LPS significantly induced macro-autophagy in both M1 and M2a lineages at 7-day polarization. Previous reports [85, 86] showed that INF-γ induced autophagy in hepatocellular carcinoma through increased LC3A and LC3B expression. Increased autophagy activity was found to increase the phagocytosis of *Mycobacterium tuberculosis* by the INF-γ signaling pathway [87]. IL-4 induced macro-autophagy in antigen-presenting B cells and is linked to asthma pathophysiology [88]. Finally, it is noteworthy to mention that IL-4 boosted autophagy induction to form LC3A and LC3B aggregates.

Our results show that the 14-day polarization resulted in the loss of arginase expression and increased autophagy-related gene expression Atg16L1-1. Since arginase 1 is a phagocytic marker for M1 and M2a, loss of expression of Arg-1 indicates loss of activation in M1 and M2a lineages at 14-day polarization [89]. Interestingly, Atg16L1-1 alpha and Atg16L1-3 gamma variant showed an increase in M2a 7-day poloarization than in M2a 14-day polarization. These studies suggest that high autophagy activity at 14-day polarization can attenuate arginase 1 expression. However, previous reports [90] show that autophagy is required for arginase 1 expression in alternatively activated M2a at 7-day polarization.

Phagocytosis assay was performed to test the ability of M0, M1, and M2a cells to engulf heat-killed *E. coli* bacteria. Interestingly, M0 (IL-6 + /CD68 +) and M1 (IL-6 + /CD68 +) cells showed significant phagocytic activity. However, M2a (Arg-1 + /CD68 +) cells showed decreased phagocytic activity. Several studies reported autophagy induction altered macrophage polarization and altered M2a phagocytic function [93–95].

As mentioned earlier, Atg16L1 is the most important hub protein in macro-autophagy and macrophage polarization. Overexpression of Atg16L1-3 and Vamp7 in M2a at 7 days of polarization and the increased number of cytoplasmic pre-autophagosomes suggest that Atg16L1 is essential for IL-4-induced macro-autophagy in M2a (Arg-1 + /CD68 +) cells. Also, autophagy induction decreased the phagocytic ability of M2a (Arg-1 + /CD68 +) cells.

To better understand the interplay between autophagy and phagocytosis, we blocked the autophagosome and lysosomal fusion with autophagy inhibitor bafilomycin A as previously described [96]. Our results indicate that bafilomycin increased CD68 and arginase 1 expression in M0, M1, and M2a lineages, while autophagy induction decreased phagocytosis of M2a but not M1. Other studies reported bafilomycin-induced autophagy inhibition and the knockdown of autophagy-related protein Atg5 promoted M2 polarization [97].

Other studies [98] reported that autophagy inhibition by 3-MA (autophagy inhibitor) increased the phagocytic ability of macrophages and rescued mice from methicillin-resistant *Staphylococcus aureus* (MRSA) bacterial infection. Also, Atg16L1 mutation increased the phagocytosis ability of monocytes isolated from Crohn’s disease patients [99]. Therefore, we suggest that Atg16L1 might serve as a therapeutic target for the treatment of altered phagocytosis-related diseases such as bacterial infection, inflammation, lupus nephritis, and cancer. Further studies for this target protein are needed.

## Conclusion

Our findings suggest that autophagy induction decreased the phagocytosis activity of M2a but not M1 macrophages. We also suggest that autophagy reprograms macrophage polarization (M1 and M2a) through CD68 and arginase 1 in an Atg16L1-1 and Atg16L1-3-dependent manner. These results might be potentially beneficial for further investigation as therapeutic targets for immunotherapy in different autoimmune disorders where macrophages play an important role in disease pathophysiology.

## Supplementary Information

Below is the link to the electronic supplementary material.

Supplementary file1 (DOCX 7208 KB)

## Data Availability

All data generated or analyzed during this study are included in this published article (and its supplementary information files).
