# Supplementary material for: Autophagic reprogramming of bone marrow–derived macrophages
Source: Immunol Res. 2022 Dec 1;71(2):229–46. doi: 10.1007/s12026-022-09344-2 (PMC10060350; doi:10.1007/s12026-022-09344-2)
Supplement: Supplementary file 1 — Supplementary file1 (DOCX 7208 KB) [file 12026_2022_9344_MOESM1_ESM.docx]

**Supplementary data**

**In silico analysis of autophagy-related genes**

**Methods**

Autophagy related Genes

A total of 249 autophagy-related genes involved in autophagy were obtained from the public Human Autophagy Database (HADb) and verified from the HUGO Gene Nomenclature Committee (HCGN) ^36^. A protein-protein interaction network was constructed using STRING V11 online interface ^37^. The produced protein Networks Data were integrated, verified, and visualized using Cytoscape 3.8.2 software ^38^. Moreover, the 249 Autophagy Associated proteins were clustered using the “Molecular Complex Detection” (MCODE) algorithm, a Cytoscape plug-in ^39^. The Molecular Complex Detection MCODE clustering algorithm resulted in 7 clusters containing 75 genes list. To narrow down the list of the 75 genes. Two clusters with the highest scores were picked to form a new hub gene list containing 24 genes. Gene Ontology terms (GO) and pathway enrichment analyses were carried out for the 24 hub genes list using the Clue Go Cytoscape plug-in ^40^.

Macrophages Differentially Expressed Genes

Two Microarray datasets for screening differentially expressed genes (DEGs) associated with macrophages M1, M2 phase polarization were obtained from the Gene expression omnibus database GEO of the National Center for Biotechnology Information under accession numbers GSE81922 ^41^ and GSE69607 ^42^*.* Preprocessing and data quality control were performed using limma package 3.26.8. R studio ^43^ and Gene Expression Omnibus 2 Repository (GEO2R) open-source code. The data normalization and log -2 transformations were recalculated using Limma package default parameters based on the threshold of fold-change > 2.0 and P-value < 0.05 for the raw data. Adjusted p-value was performed with the default parameter in the limma package using False discovery rate (FDR < 0.05) ^44^.

The top 250 highly differentially expressed M1, and M2 polarization genes were selected as the DEGs list for M1 and M2a polarization. Functional annotation and pathway enrichment analysis of the M1 & M2 DEGs list was performed using the Clue Go plug-in Cytoscape 3.6. The enriched pathways appeared according to their significance p-value < 0.05 using the Enrichment/depletion two-sided hypergeometric test ^45^, using Bonferroni step down test to show the highest significance in the enrichment network*.*

The DEGs list of M1 and M2 curated from GSE81922 and GSE69607 Microarray Data were then used as protein input for STRING 11.V to construct a protein-protein interaction network. The parameters of prediction were set after removing the test mining to reduce the error in prediction. The confidence interval was set to a high confidence interval (0.7). The top DEGs were clustered using the MCODE algorithm plug-in on Cytoscape to extract the Hubs in the network. Clustering resulted in finding 18 Hub genes.

Common transcription factors prediction

In silico prediction was performed to obtain the common regulon and transcription binding motifs that regulate both autophagy genes (24 genes) and M1, M2 polarization DEGs list (18 genes). The I-regulon ^46^ Cytoscape 3.8.2 plug-in was used for this prediction, using the default parameters with a maximum false discovery rate (FDR) on motif similarity of 0.001. Prediction via I regulon resulted in 12 Gene regulatory transcriptional networks. Which then were manually verified using TRANSFAC and JASPAR databases.

Regulatory miRNAs Prediction for Gene-Transcription

Three databases, Target scan Mouse 7.1 ^47^, miRBase ^48^, and Network Analyst ^49^*,* were studied to predict the common regulating upstream miRNAs that link between autophagy and macrophage polarization. We found 8 predicted miRNAs to have a common link between Autophagy and Macrophages Polarization in the Phagocytosis process.

**Results**

**In silico analysis of autophagy-related genes**

We used a Network-based systems biology approach (Figure 1S) to model the interplay between the complex signaling pathways of autophagy and macrophage polarization. The analysis of the different databases identified common significantly enriched pathways, common regulatory transcription factors, and upstream miRNAs that co-regulate both transcription factors and Atgs & M1-M2-DEGS.

Genetic and protein regulatory networks.

The protein-protein interaction network of autophagy-related genes in mice was constructed by STRING V11 online interface ^37^. The number of nodes was 221, the number of edges was 638, the average node degree is 5.7, and the average local clustering coefficient was 0.42 with a protein-protein interaction enrichment p-value < 1.0 e-16 (Figure 2S).

A total of 7 clusters ranked from the highest score to the least score. Networks with the highest scores were selected for pathway enrichment with Clue Go. The highly significant pathways were Cellular response to nitrogen starvation, Apelin signaling pathway, selective autophagy, and autophagosome assembly (Figure 3S). Biological processes were visualized in terms of node size and log10 p-value. The highly significant biological processes identified are autophagy, lipoprotein metabolic processes, cellular response to nitrogen levels, and autophagosome assembly. The autophagosome assembly biological processes indicated the importance of the proteins responsible for autophagosome assembly, such as ATG16l1, LC3 a & b, and ATG7 (Figure 3S).

String predicted the interaction between these proteins forming a protein-protein interaction network; the significance of this network is (p-value < 1.0e-16) (Figure 4S).

Using the MCODE algorithm clustering plug-in, 7 clusters were generated. Cluster 1 contained 15 genes with a score of 14.7 and BECN1 as a hub gene. Cluster 2 (8.22) and AMBRA1 as a hub gene. Cluster 3 (4.087) and EGFR as a hub gene. Cluster 4 (score 3) and RELA as a hub gene. Cluster 5 (score 3) and DNAJB1 as a hub gene. Cluster 6 (score 3) and MYC as a hub gene. Cluster 7 (score 3) and MAPK 9 as a hub gene. These clusters identified the hub proteins in the protein-protein interaction network, which are the proteins that have the most interactions (binding sites) for other non -hub proteins (Figures 6S).

The Clue go analysis showed the significant functional go terms from KEGG pathways (Figure 3S). Significance in terms of p-value < 0.05 and node size and nodes are linked according to their kappa score level (≥ 0.3).

M1-M2 Macrophages Polarization Networks

The String interface generated a protein-protein interaction network for macrophages M1 –M2 gene set of 250 DEGs from the datasets (GSE69607 & GSE81922). The resulting network (Figure 7S) showed the number of nodes was 218, the number of edges was 160, and the average node degree is 1.47. The Average local clustering coefficient was 0.304. The significance of protein-protein interaction enrichment was high (p-value < 0.001(1.03e-14) with the confidence of 0.4, which means a high confidence degree of interactions presented in the network.

The MCODE Clustering algorithm clustered the top 250 gene set of DEGs genes to define the network areas with the highest interaction. And, to determine the gene hubs in the generated 6 clusters and the 6 hub genes identified. Clusters were identified with CXCL3, ATP6V1B2, MITF, IFG1, IRAK2, and LPIN2 as hub genes (Figures 8S).

These highly enriched pathways in M1 & M2 polarization were significant in terms of node size. Also, KEGG pathway enrichment (P-value is <0.05) involved pathways such as; regulation of wound healing, IL7 signaling pathway, TNF signaling pathway, keratinocyte migration, and FOXO signaling pathway (Figure 3S).

LC3A & B and Atg7 proteins are autophagy-related proteins that are also enriched in autophagosome assembly. Atg16L1 is an autophagy-related protein and has a role in autophagosome assembly.

Common Transcription Factors Regulatory Networks for both Autophagy Associated Genes and M1-M2 DEGs Clusters

We noted that Atg16L1, MAP1LC3A&B, IL6, and Atg7 were the most abundant genes in the 12 predicted transcriptional factor regulatory networks. Predicted common transcription factors that regulate both Autophagy genes clusters and M1 & M2 polarization genes formed 6 clusters: Homez, MLXipl, Mycs, Rel, Smad1, and Stat4 transcription factors. These are common factors that regulate both autophagy and M1 & M2 polarization genes (Figure 8S).

Atg16L1 protein tertiary structure

The tertiary structure of Atg16L1was predicted using the amino acid sequence of each isoform of Atg16L1 after multiple sequence alignment using the SWISS Prot online tool (Figure 9S).  The predicted models were numbered according to their quality using a global quality estimate (QMEAN). Isoforms Atg16L1-1 and Atg16L1-3 were selected for further studies due to the structural similarity between Atg16L1 -1 (Alpha) and Atg16L1-3 (Gamma) (Figure 10S). We examined their mRNA transcriptional level in M0, M1, and M2a lineages at day 7 and day 14 polarization. The Atg16L1-1 variant showed a significant fold change at day 7 and day 14 polarization. There was an expression variation between Atg16l1-1 and Atg16L1-3 gene variants in both M1 and M2a lineages at D7 and D14 polarization (Figures 9S & 10S).


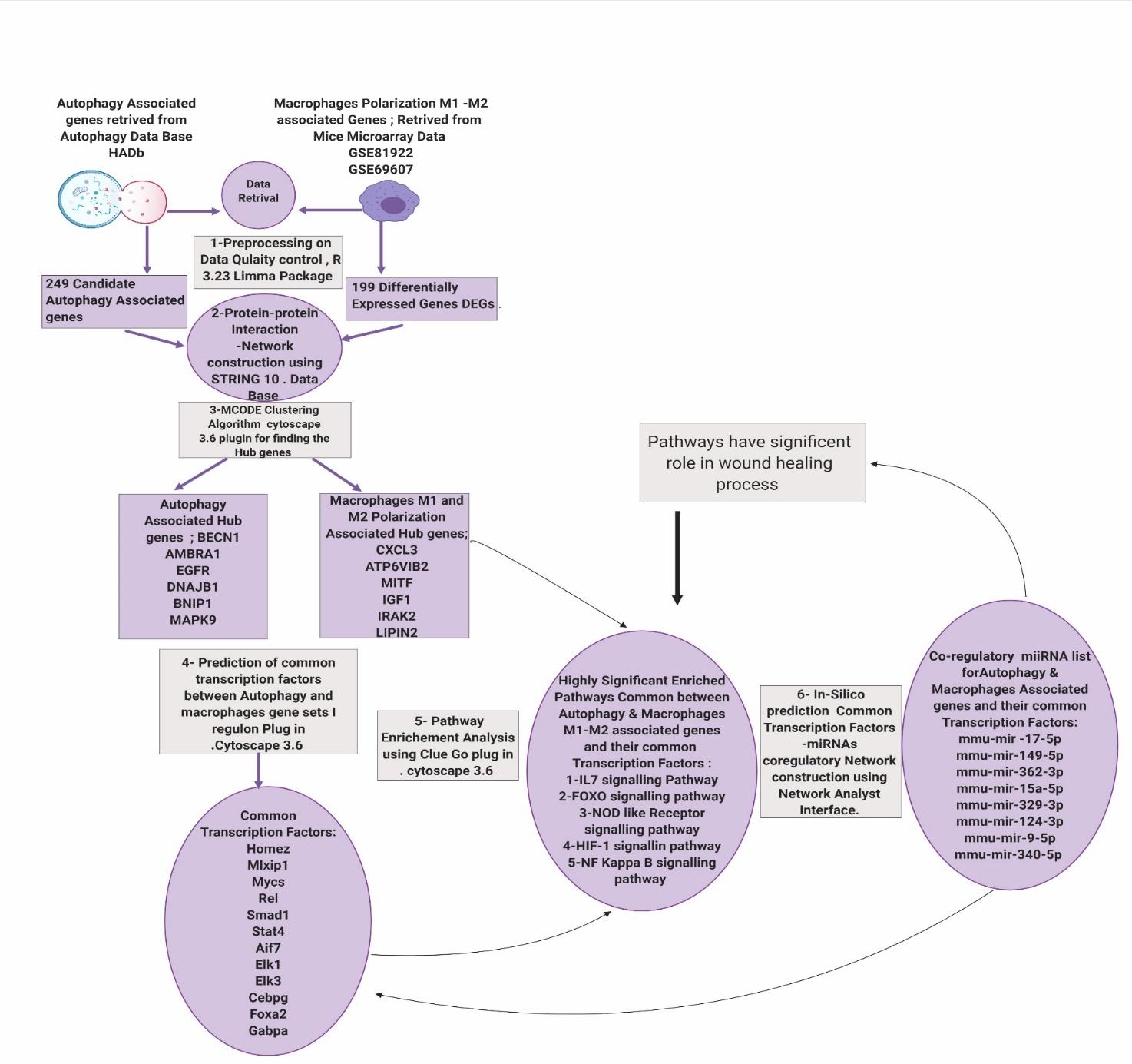



**Figure 1S: Representation of Systems Biology Approach.**

Autophagy-related genes (ATGs) were retrieved from the public autophagy database HADb and the gene expression data retrieved from Gene Expression Omnibus (GEO); GSE81922 & GSE69607 microarray data macrophages polarization 250 genes (DEGs). For both ATGs and DEGs gene sets, an integrated protein-protein interaction network was constructed, go terms pathway enrichment analysis was performed. Figure created with BioRender ®.

| 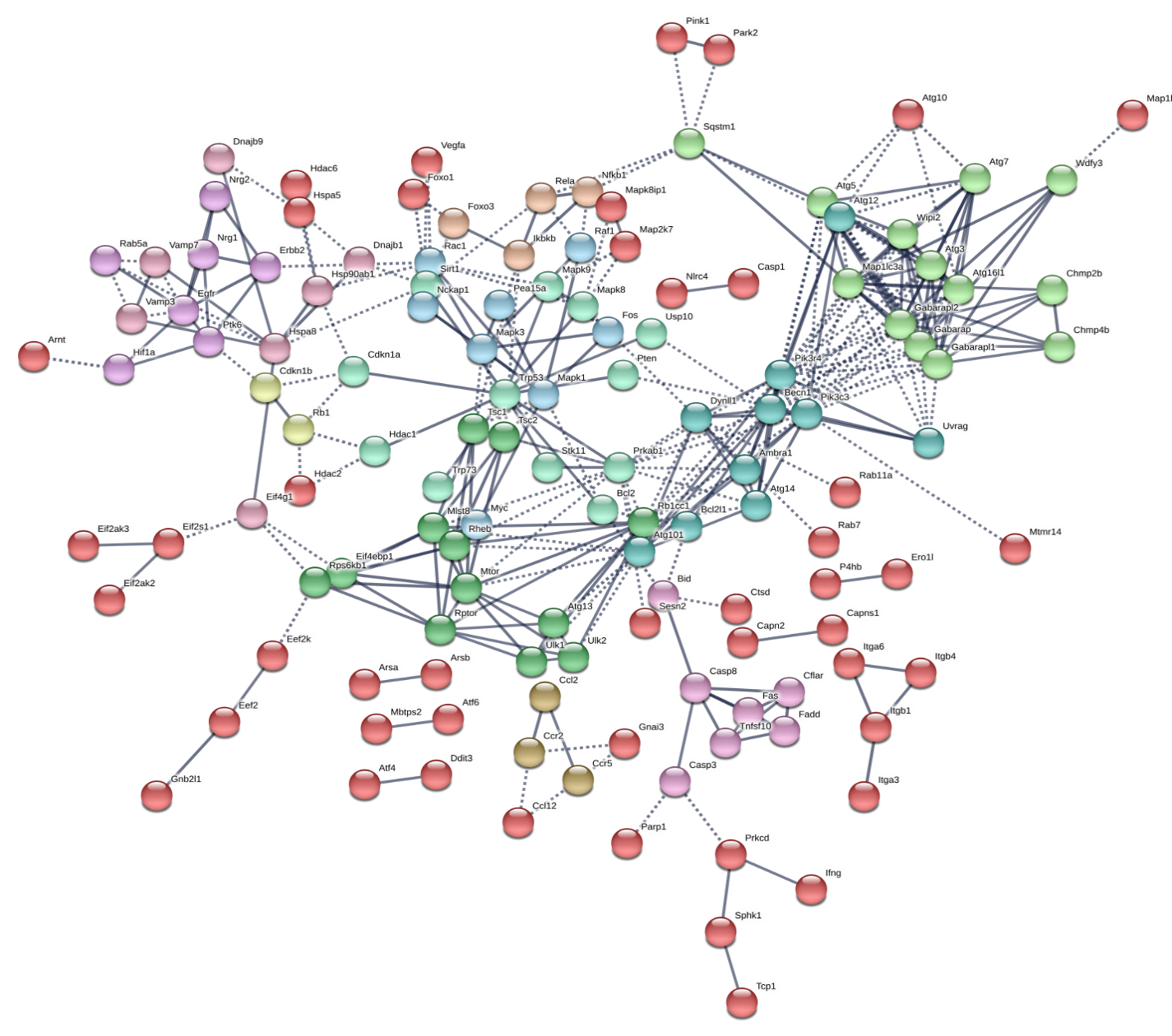 |
| --- |

**Figure 2S. Protein-Protein interaction**

STRING predicted the interaction between Autophagy-associated proteins forming a protein-protein interaction network. Thick lines mean strong interactions. Red nodes are query proteins and the first shell of interactors. The significance (confidence degree of interactions) is (p-value < 1.0e-16).

| 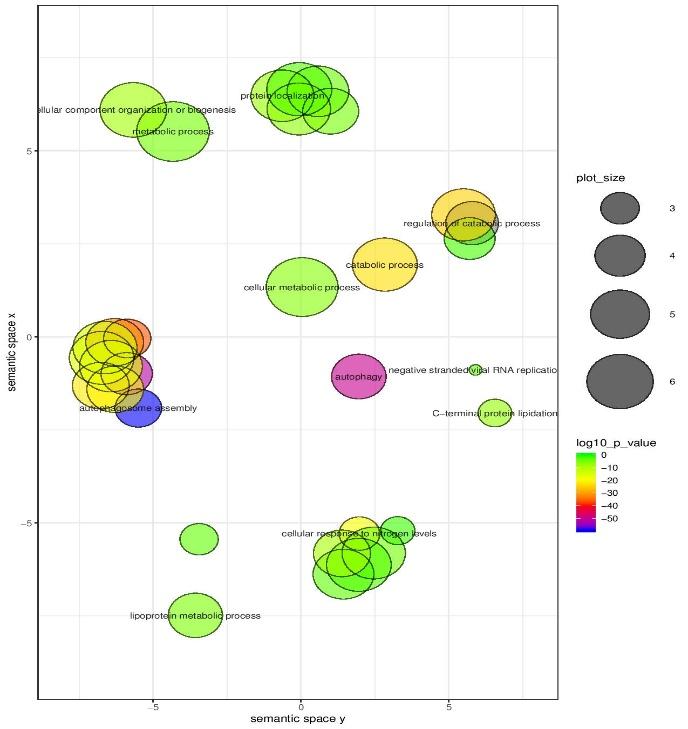 A | 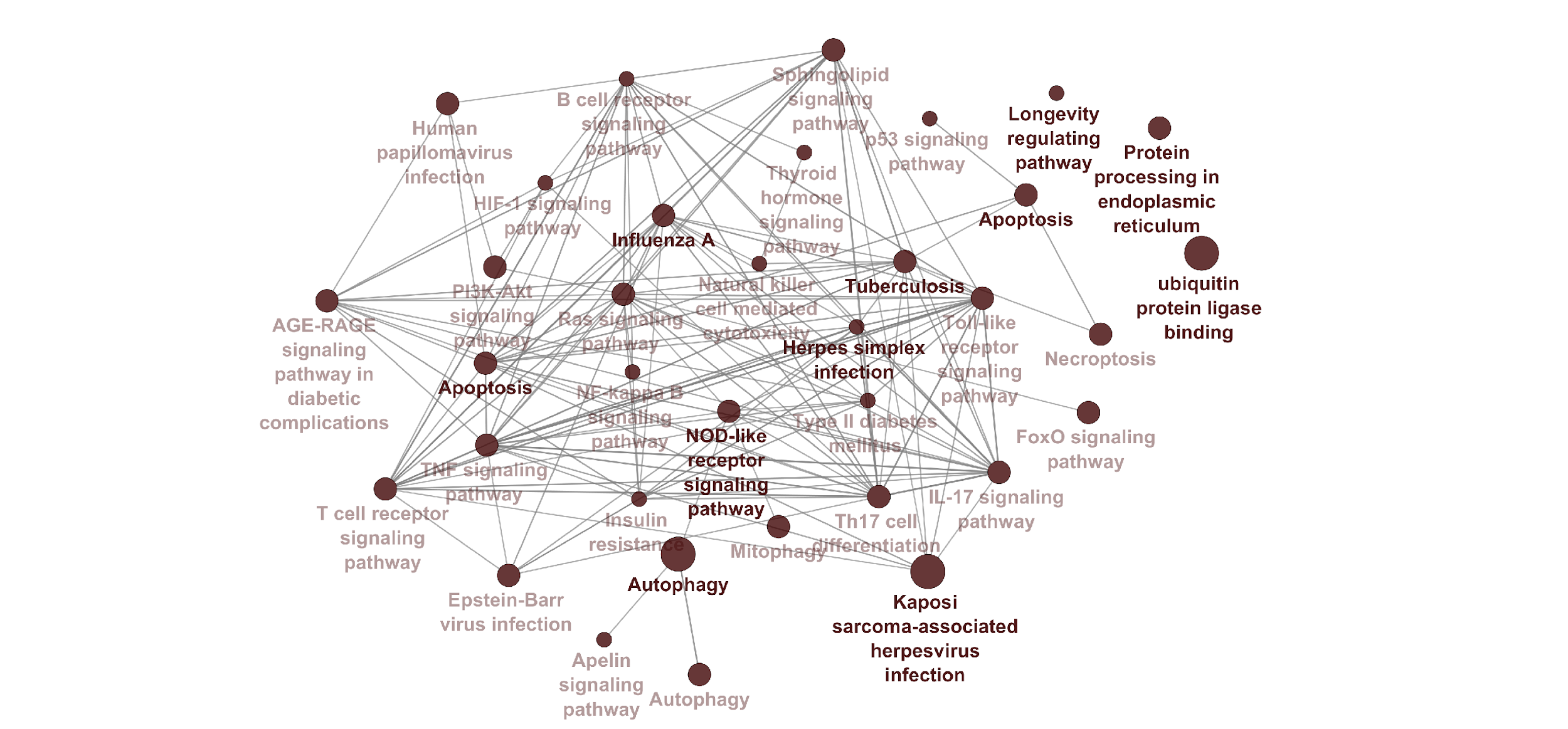 B |
| --- | --- |
| 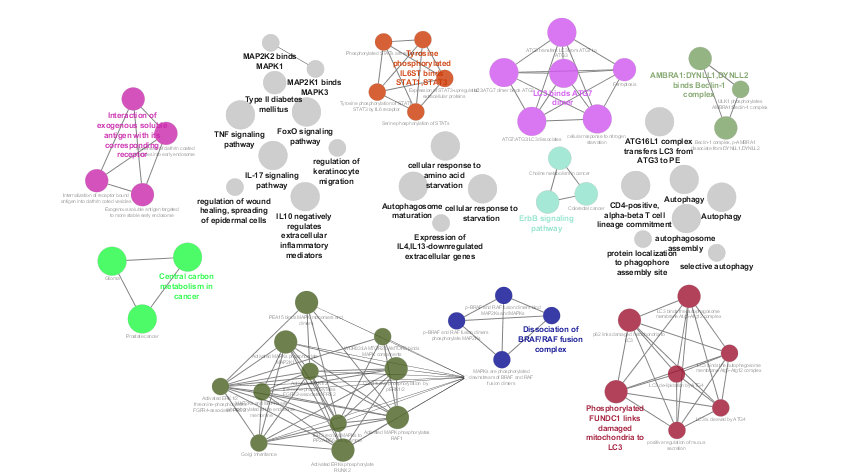C | |

**Figure 3S. Significantly enriched biological processes and Autophagy Related Proteins.**

Significantly enriched biological processes were visualized (a). The significance was set in terms of node size and log10 p-value. Autophagy, lipoprotein metabolic process, cellular response to nitrogen levels, and autophagosome assembly were identified as highly significant biological processes. Go terms KEGG pathway enrichment graph (b), significance was set in terms of p-value <0.05, and node size. Nodes were linked according to their kappa score level (≥ 0.3). Significance was set in terms of the corrected p-value. Significant cellular processes included tissue remodeling, response to interleukin -4, response to interleukin –1, Positive regulation of NF-Kappa transcription factor activity, Aging. Clue Go pathway enrichment shows highly enriched pathways in M1 and M2 polarization (C). Sic Clusters were identified (brown nodes are highly significant). Also, the KEGG pathway enrichment P-value is <0.05.

| **A**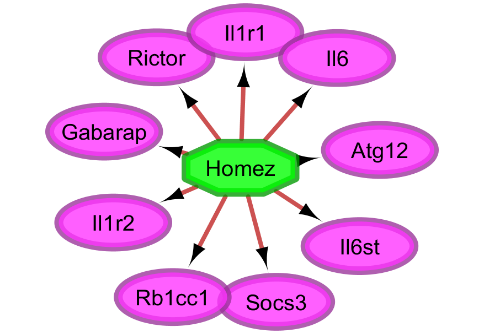 | B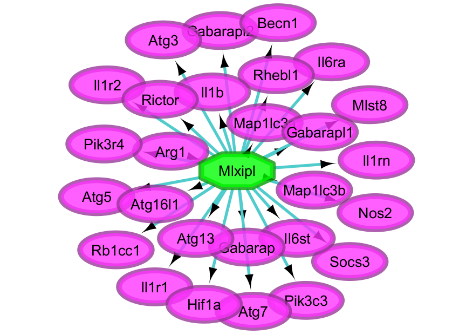 | C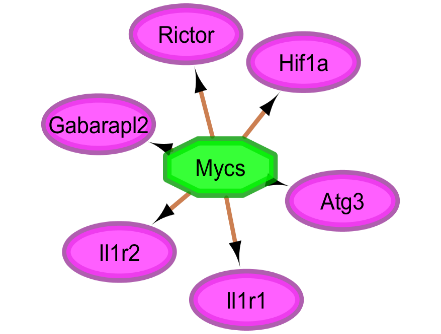 |
| --- | --- | --- |
| **D**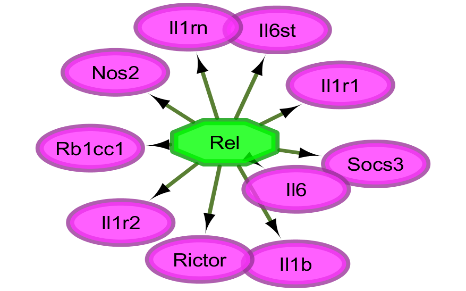 | E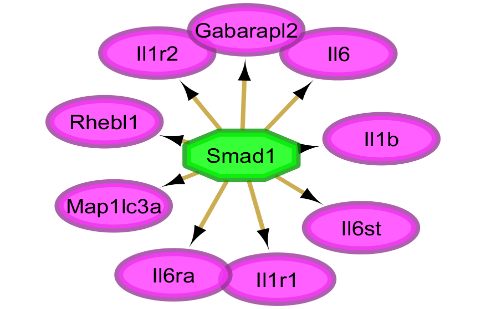 | F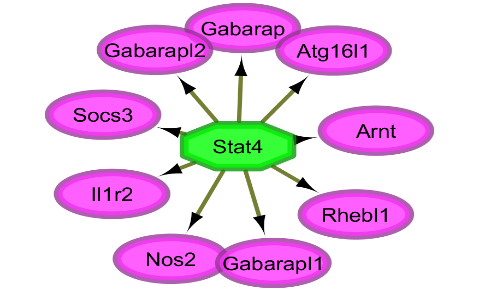 |
| **G**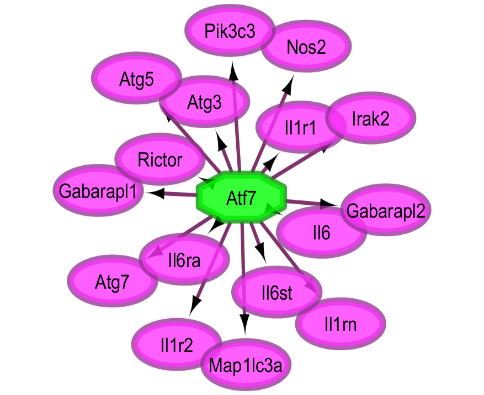 | H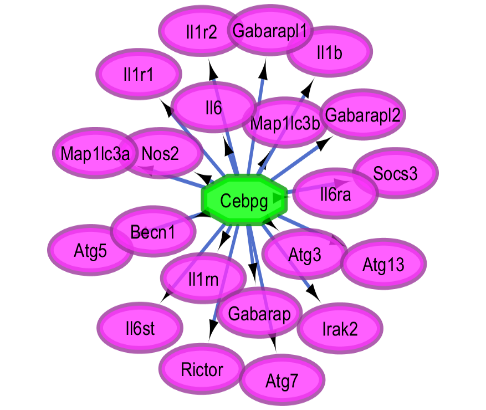 | I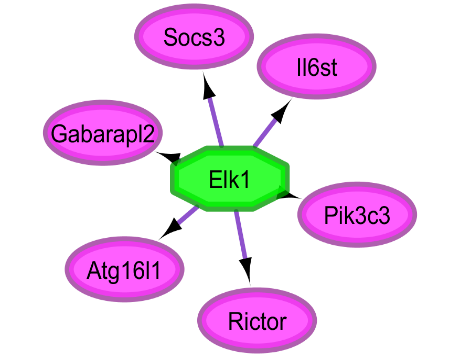 |
| **J**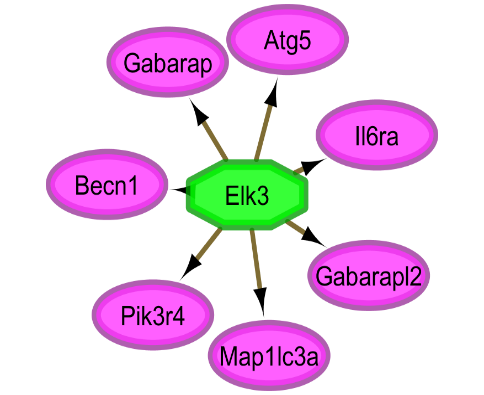 | K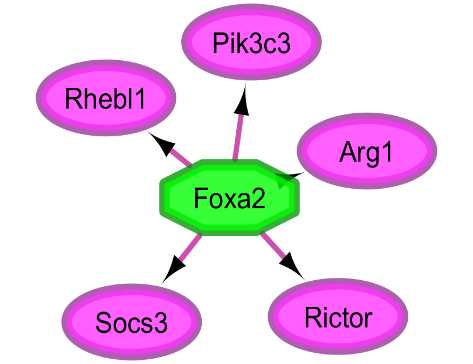 | L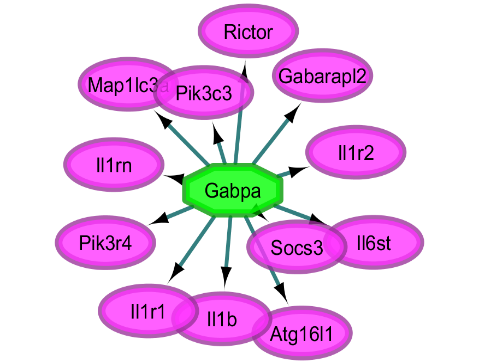 |

**Figure 4S. Cytoscape predicted common transcription factors.**

Common transcription factors regulating both Autophagy genes (8 clusters) and M1 & M2 polarization genes (6 clusters). Transcription factors are shown in green, and downstream target genes are shown in pink. Homez (A), MLXipl (B), Mycs (C), Rel (D), Smad1 (E), Stat4 (F), Atf7 (G), Cebpg (H), Elk1 (I), Elk3 (J), Foxa2 (K) and Gabpa (L) were identified as common transcription factors that regulate both autophagy and M1 & M2 polarization genes.

| **Red nodes are query proteins and the first shell of interactors**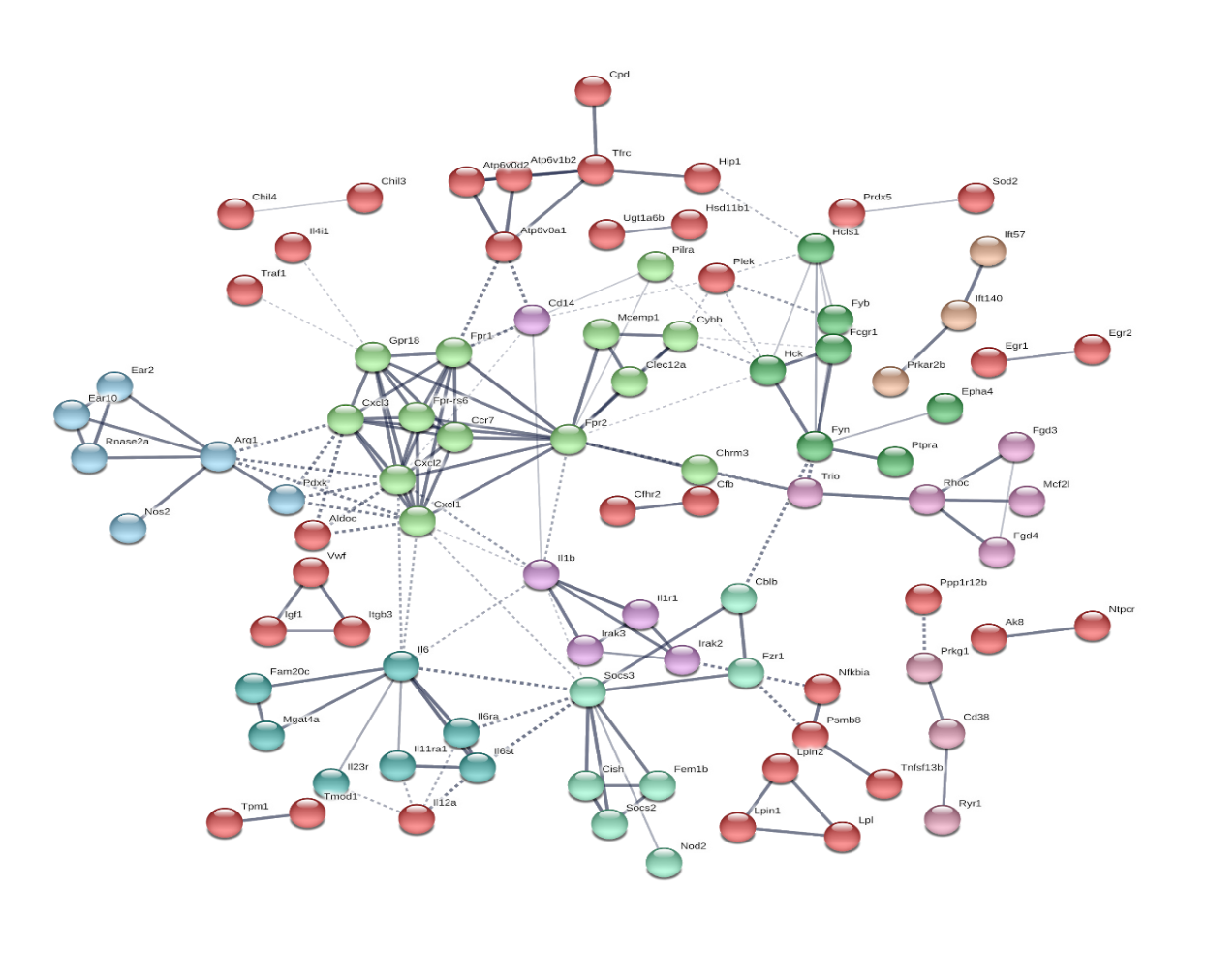 |
| --- |

**Figure 5S. String protein-protein interaction network of M1-M2 polarization.**

The network shows the number of nodes: 218, number of edges: 160, average node degree: 1.47, local clustering coefficient: 0.304, significance in terms of protein-protein interaction enrichment p-value <0.01(1.03e-14) with the confidence of 0.4. Here significance means the confidence degree of interactions presented in the network. The thick lines between nodes are based on their evidence of interaction and significant interaction.

| A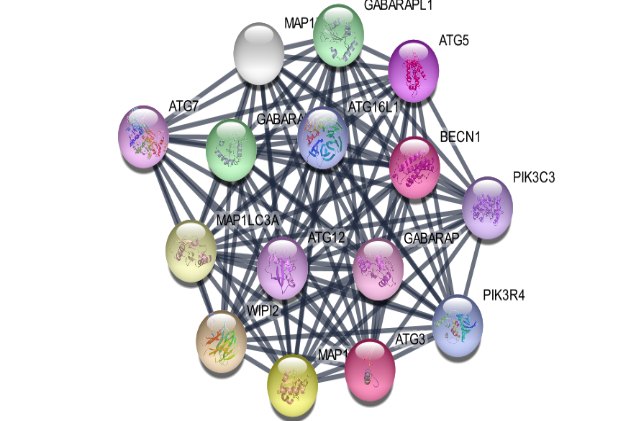 | B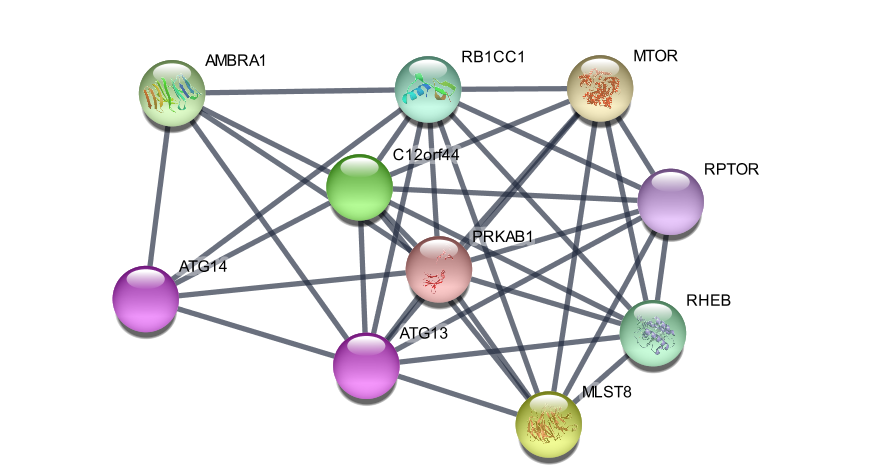 |
| --- | --- |
| C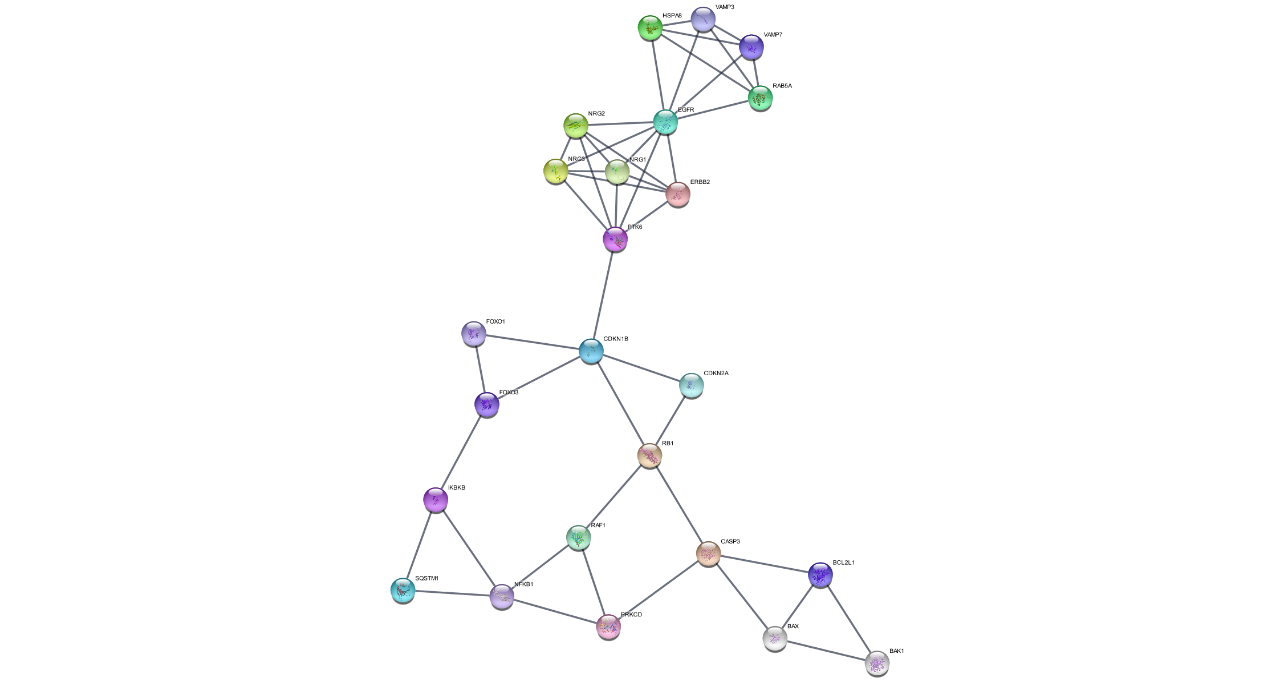 | D 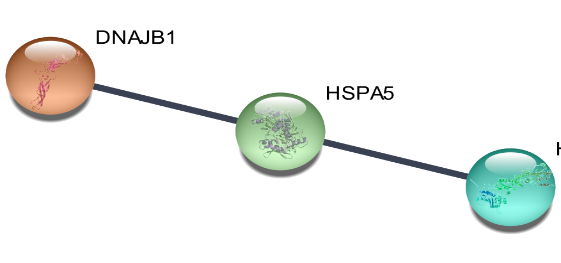 |
|  | 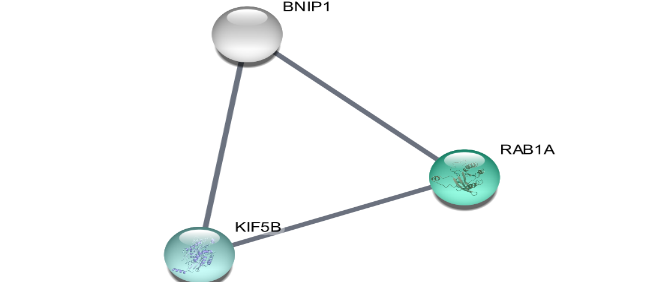  E |
| F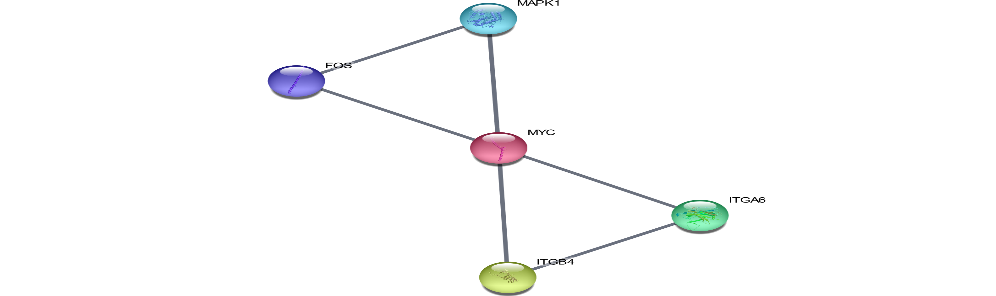 | 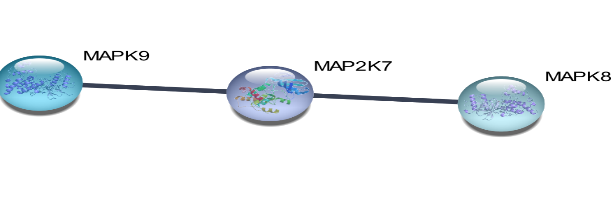  G |

**Figure 6S. Autophagy gene set & highly connected gene clusters.**

Using the MCODE algorithm clustering plugin, 7 clusters were generated. A) cluster 1 contained 15 genes with a score of 14.7 and BECN1 hub gene. B) Cluster 2 score of 8.22 and AMBRA1 hub gene. C) Cluster 3 score of 4.087 and EGFR hub gene. D) cluster 4 score 3 and RELA hub gene. E) Cluster 5 score of 3 and DNAJB1. F) Cluster 6 score of 3 and MYC hub gene) cluster 7, score 3, and MAPK 9 hub gene. The importance of clustering is sorting the hub proteins in the protein-protein interaction network.

| **A**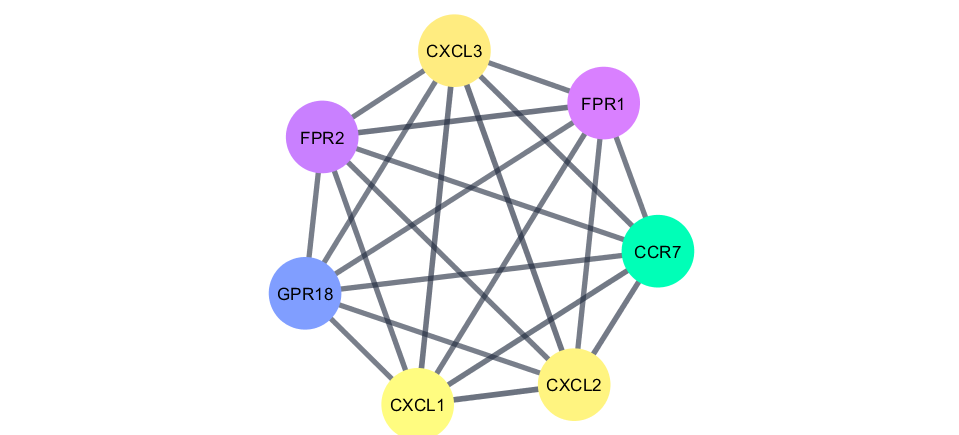 | **B**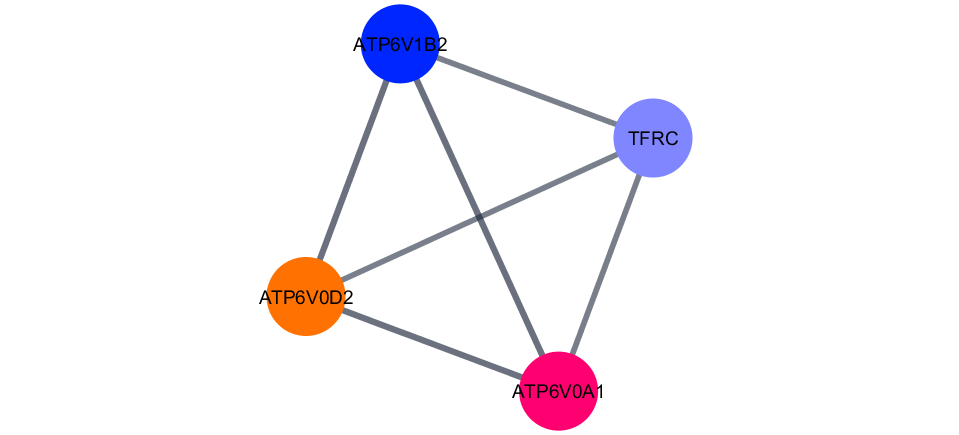 |
| --- | --- |
| **C**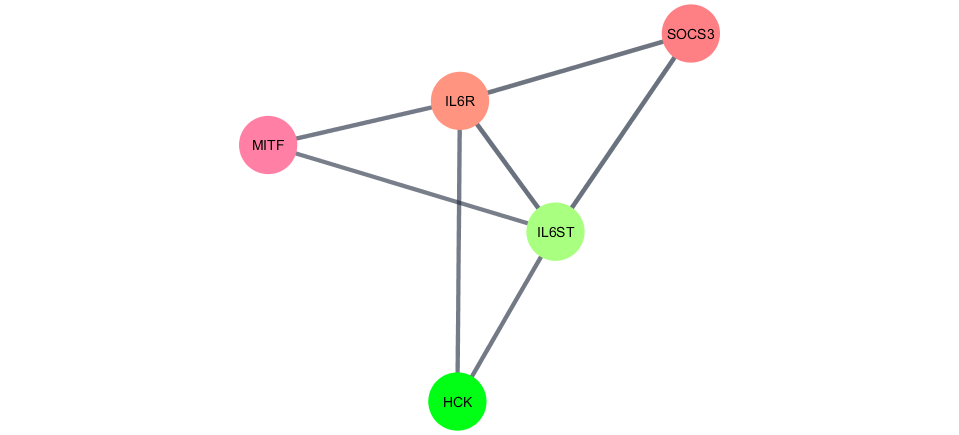 | **D**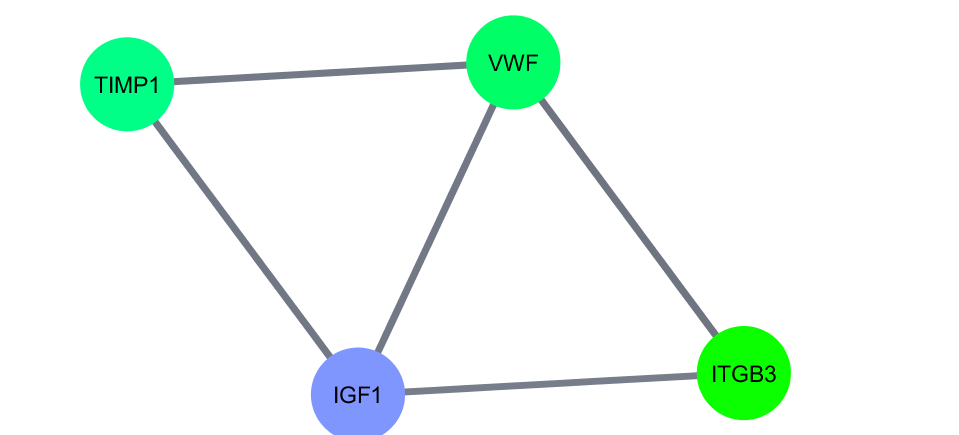 |
| 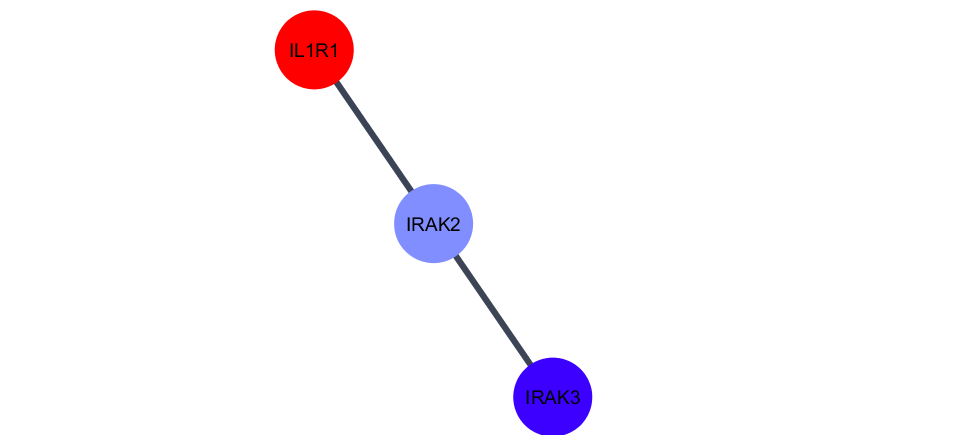  **E** | 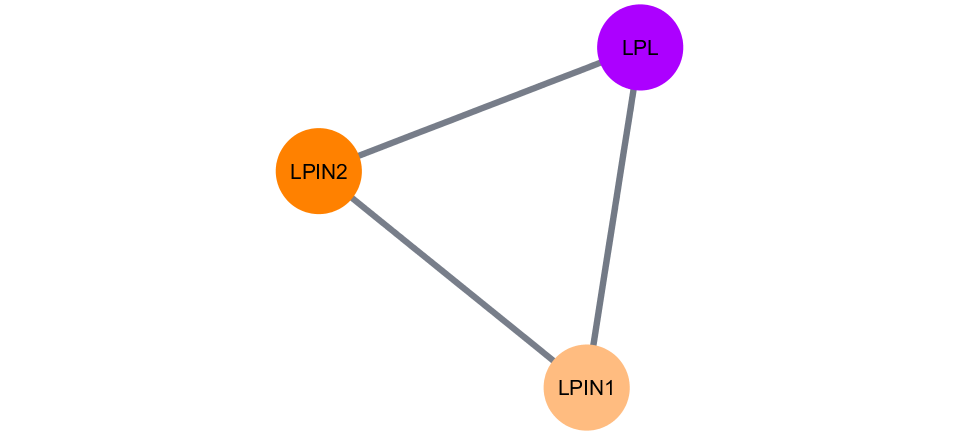  **F** |

**Figure 7S. MCODE clustering of differentially expressed genes (DEGs) in M1 & M2 Macrophages.**

The MCODE clustering algorithm Cytoscape identified six clusters with 6 hub genes as follows: A) Cluster 1 with a score of 7 and CXCL3 hub gene. B) Cluster 2 with a score of 4 and ATP6V1B2 hub gene. C) Cluster 3 with a score of 3.5 and MITF hub gene. D) Cluster 4 with a score of 3.33, IFG1 is the hub gene. E) Cluster 5 with a score of 3, and IRAK2 as a hub gene. F) Cluster 6 with a score of 3, and LPIN2 as a hub gene.

| 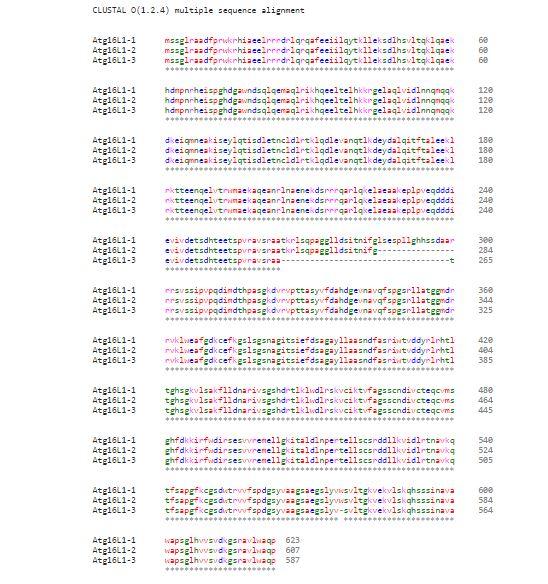  **A- Multi sequence Alignment for Atg16L1 Isoforms** | **B- Structure of Atg16L1 isoforms** 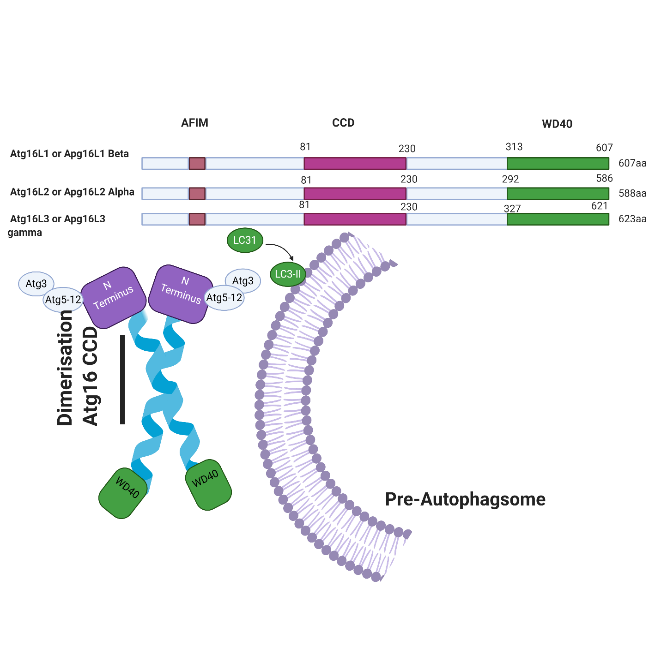 |
| --- | --- |
|  | **C- Atg16l1 – lysosomal homotypic fusion** 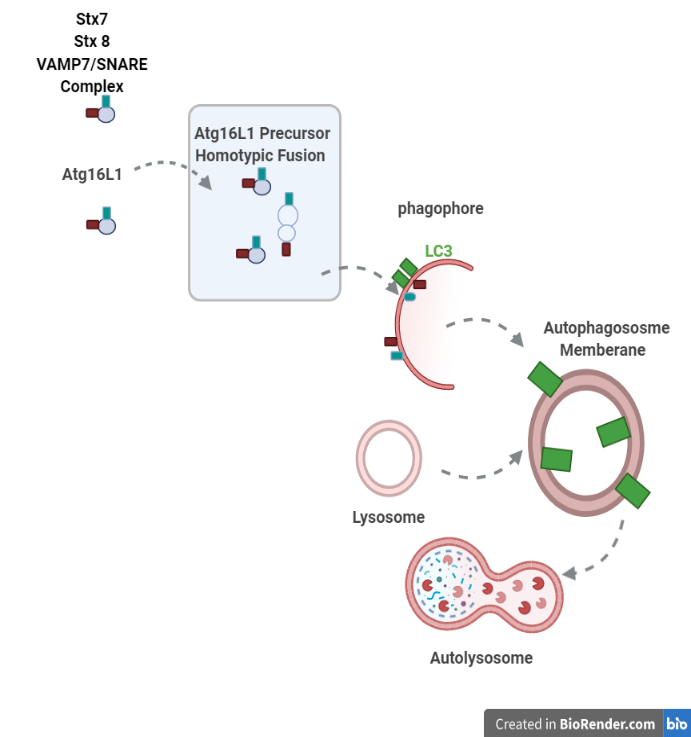 |

**Figure 8S. Computational Multi-Sequence Alignment of Atg16L1 -1, Atg16L1-2, and Atg16L-3.** (A) Multi-sequence Alignment using Clustal software showed a high amino acid sequence similarity among Atg16L1-1 or alpha and Atg16L1-3 or gamma. It also describes the similarity in the N- terminal domain in both isoforms Atg16L1-1 and Atg16L1-3 (B) diagram described the anatomical structure of the Atg16L1 protein with its 3 isoforms and its N-terminal domain. N terminal domain is crucial for binding with lysosomal complex protein Vamp7 at (C). Figures B & C created with BioRender ®.

| A-Atg16l1-5 Isoform, WD40 domain 311 aa 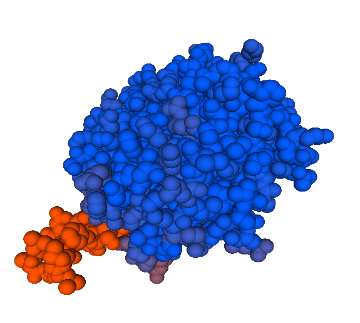 | 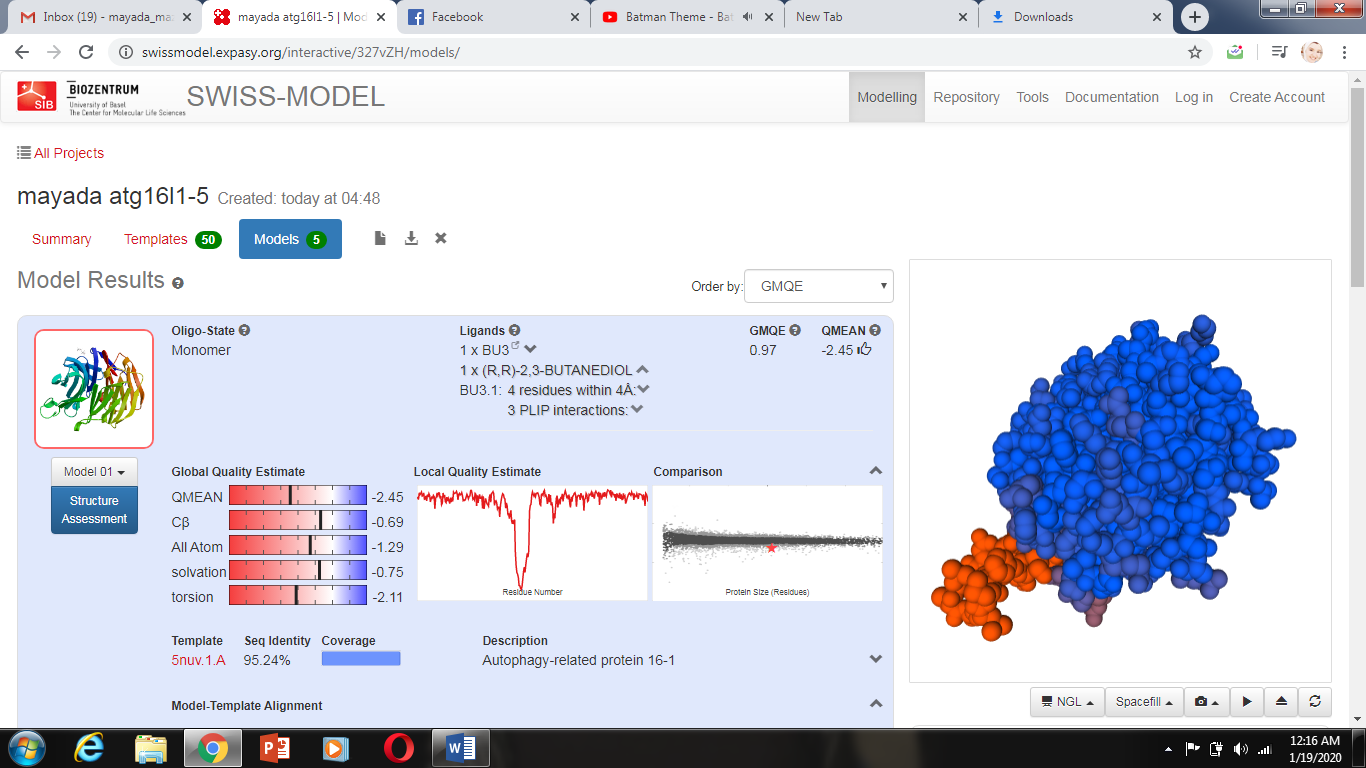 |
| --- | --- |
| 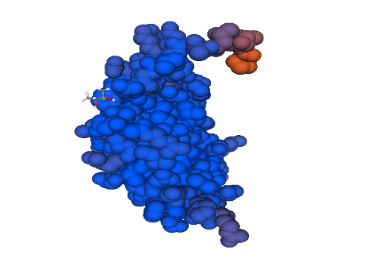  B - Atg16l1-4 Isoform, WD40 domain 126 aa | 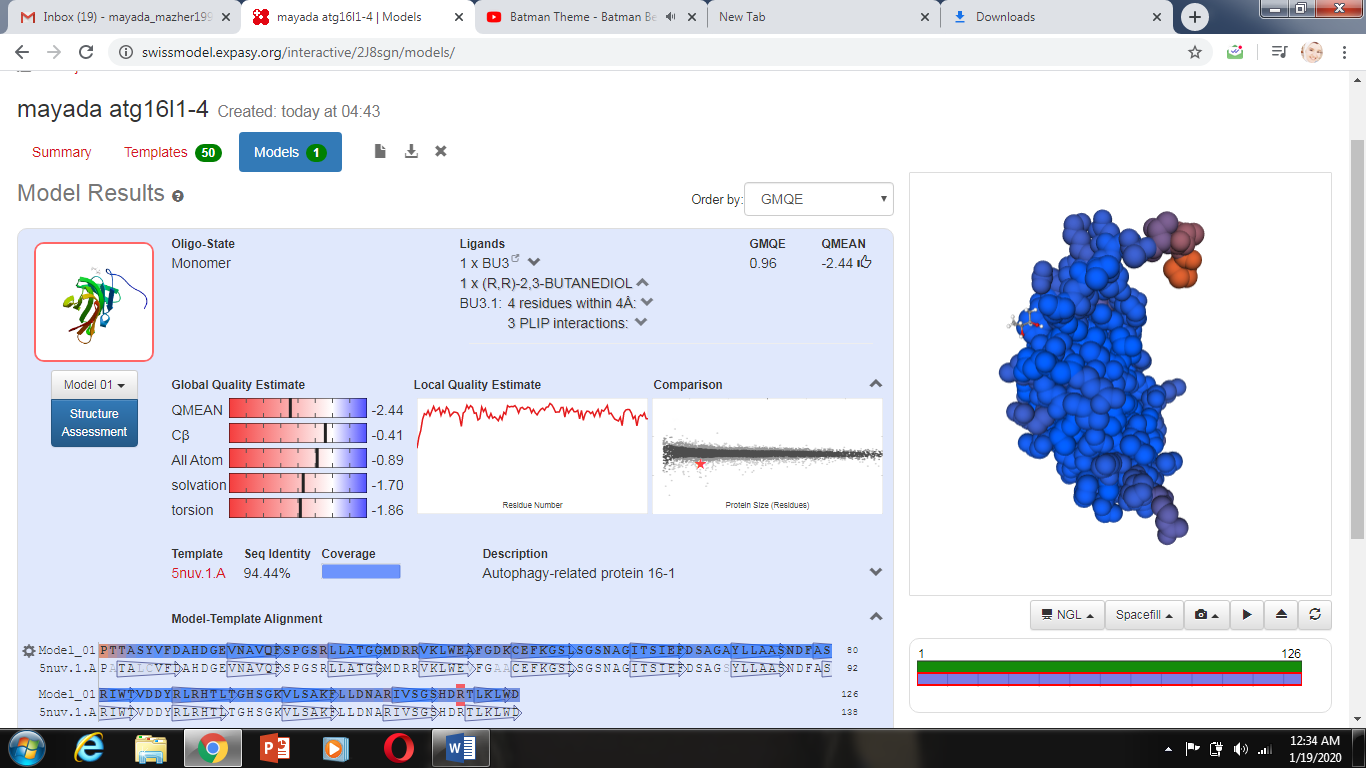 |
| C-Atg16l1-3 γ isoform, WD40 domain 295 aa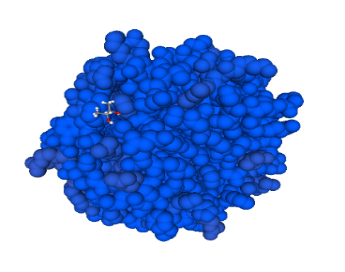 | 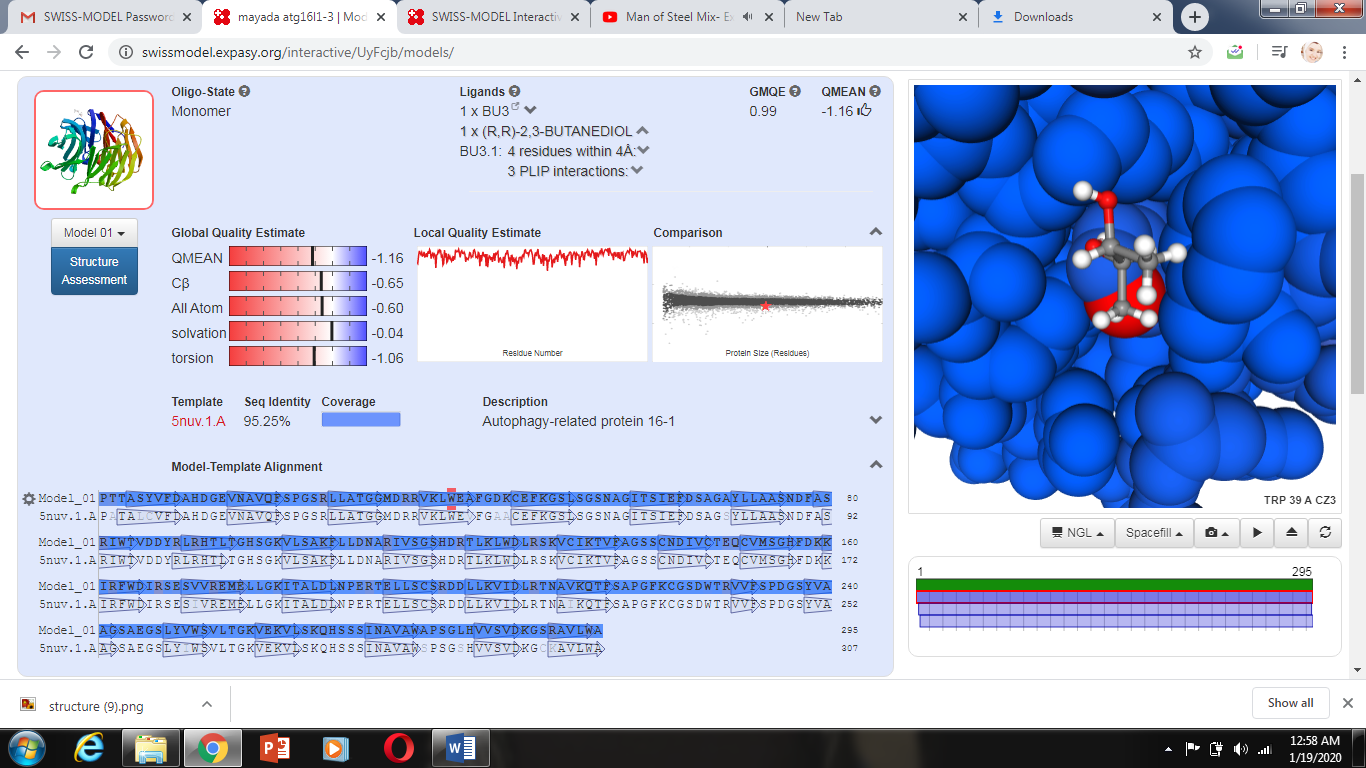 |
| D-Atg16L1-2 β isoform, WD40 domain 296 aa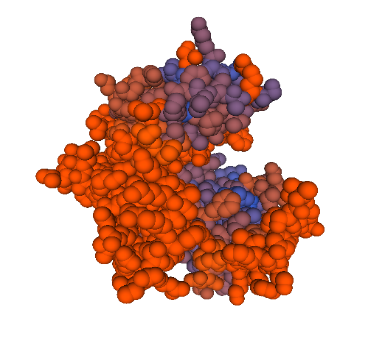 | 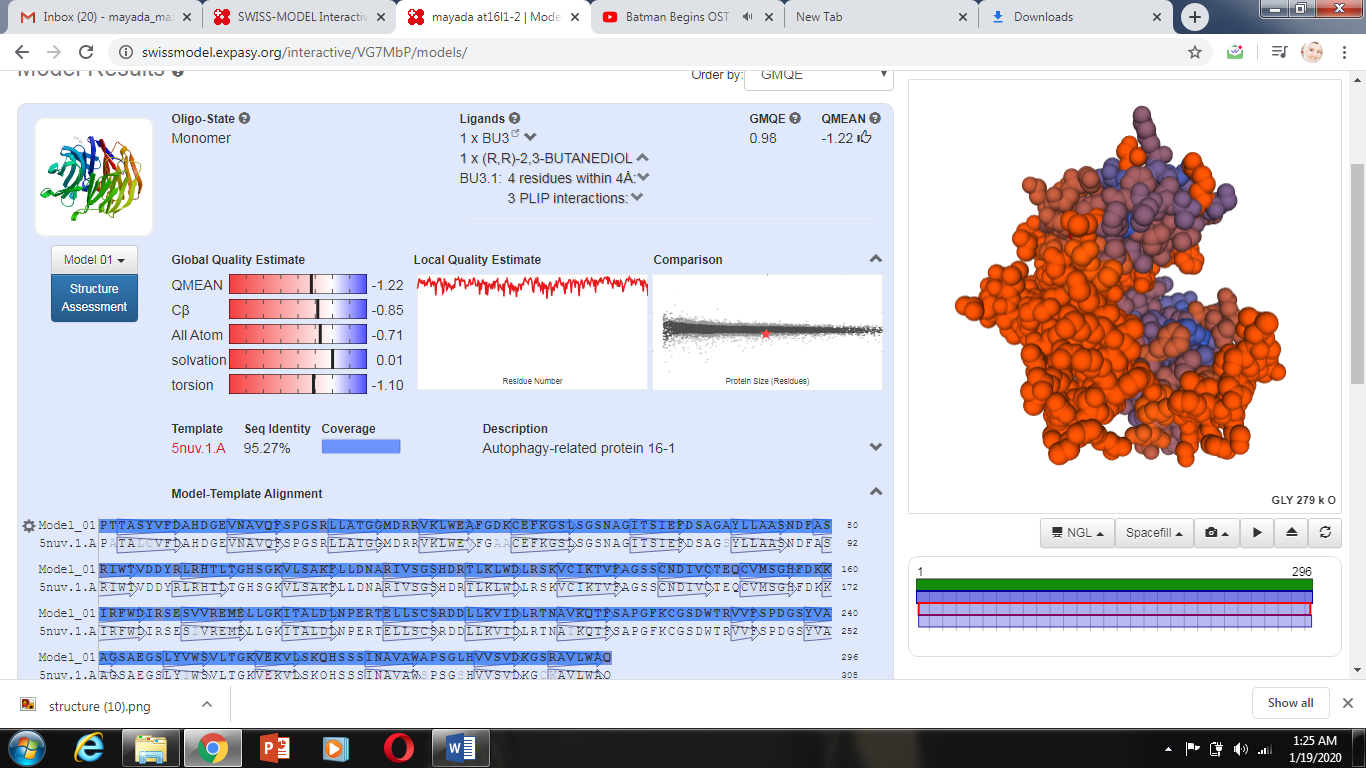 |
| E- Atg16L1-1-α isoform, WD40 domain 296 aa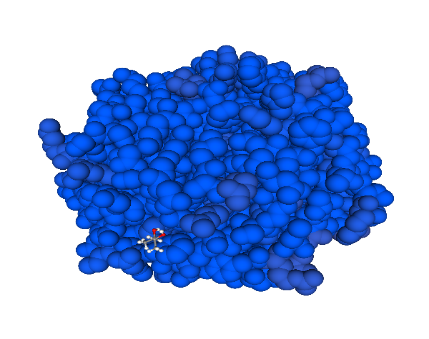 | 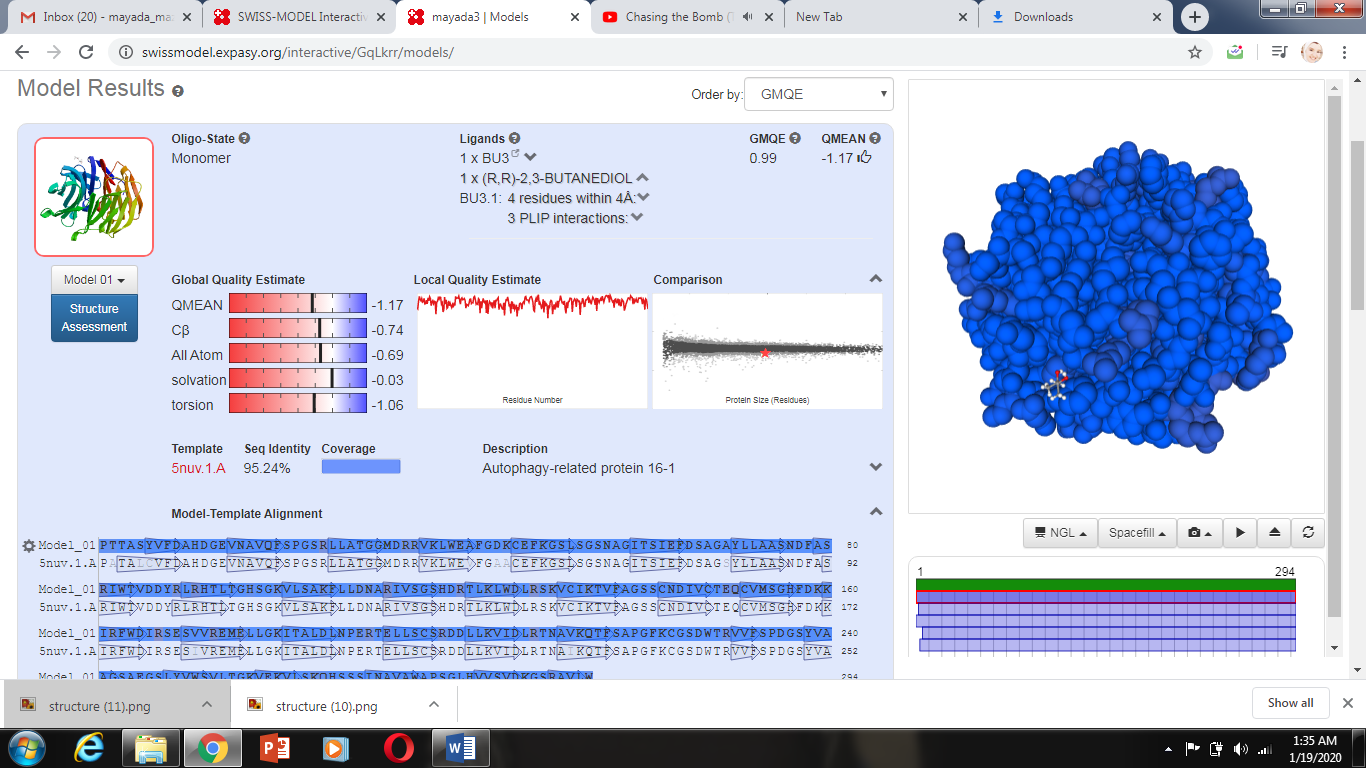 |

**Figure 9S. The predicted tertiary structure of Atg16L1 isoforms.** The tertiary structure was predicted using the amino acid sequence of Atg16L1 (5 isoforms) on the Swiss model plugin. Then predicted models or structures were numbered according to their quality using the global quality estimate. QMEAN is one of the indicators of structural similarity and model quality. Structure (C) and (E) showed the higher 3d structure similarity for (C) Atg16L1-3 QMEAN (-1.16) and for (E) Atg16L1-1 QMEAN value is (-1.16).

| **A**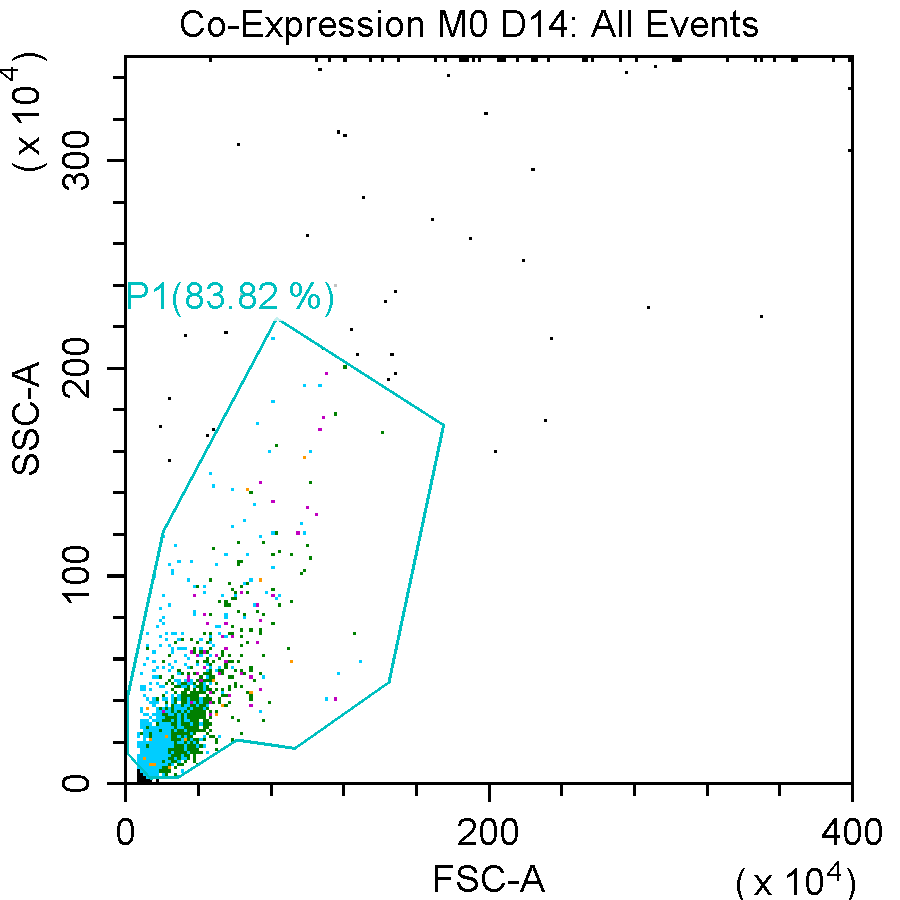 | **B**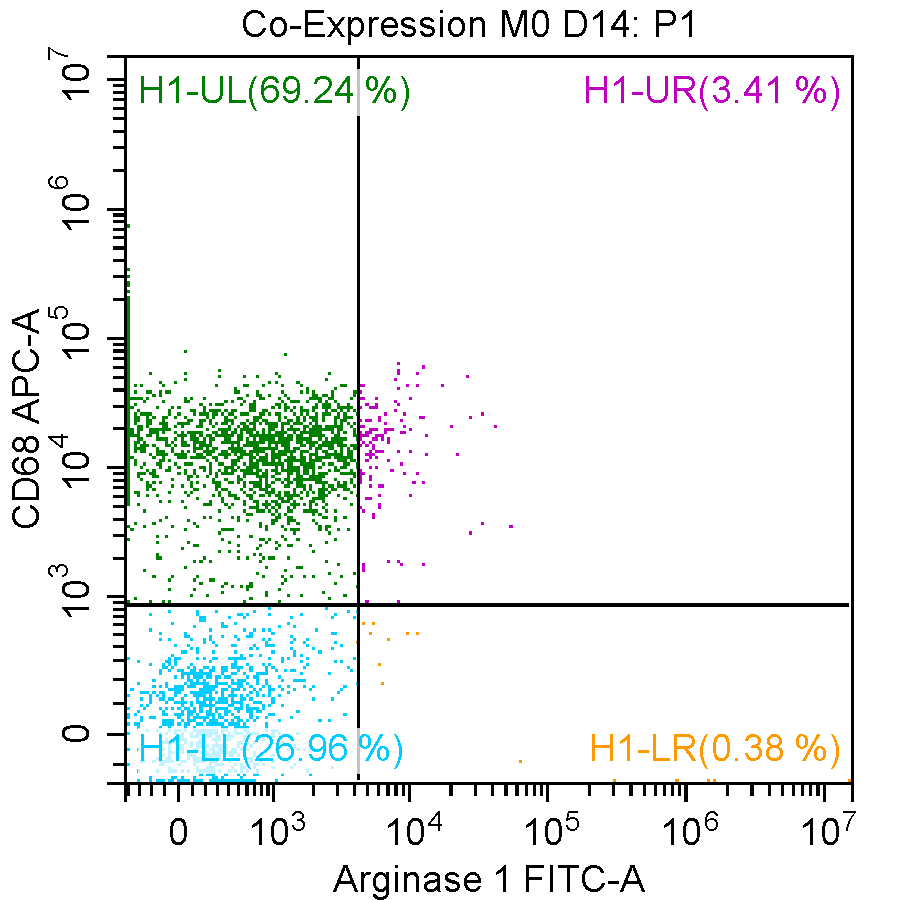 | **C**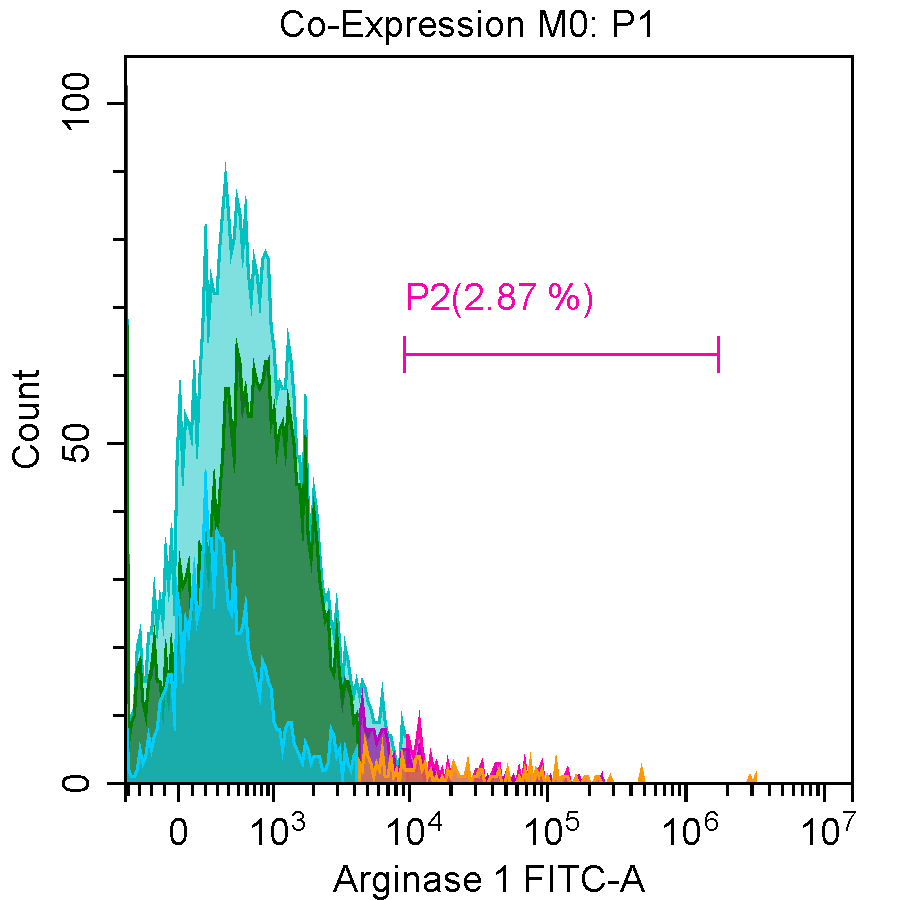 | **D**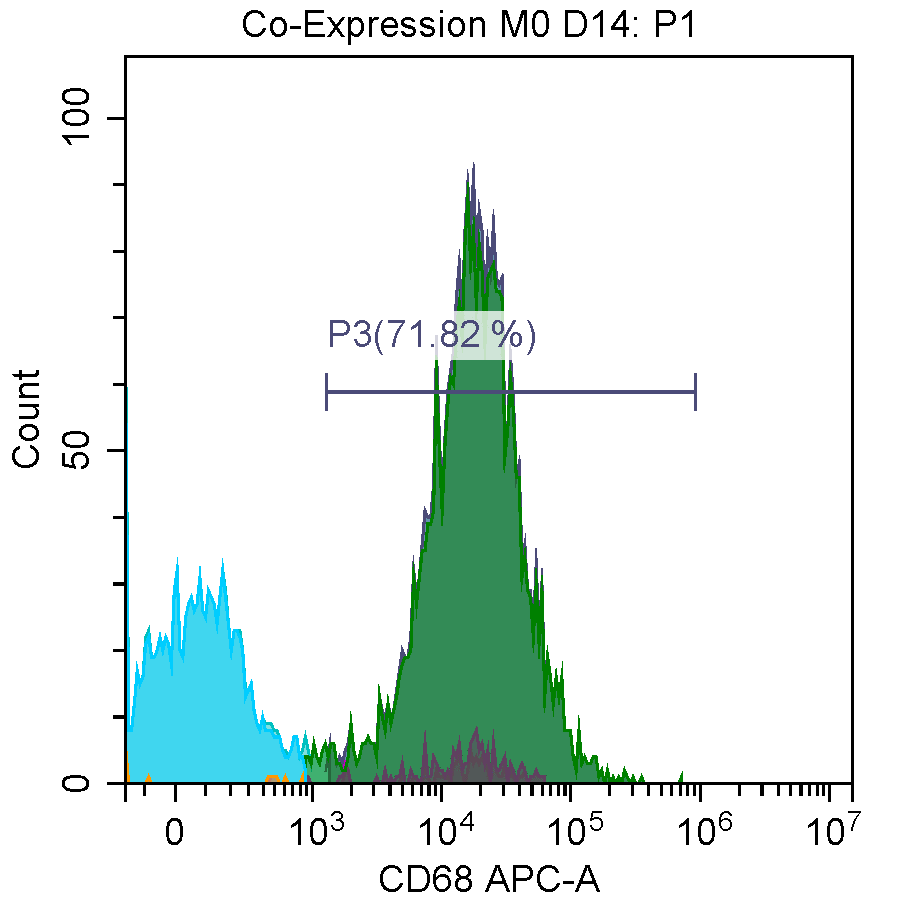 |
| --- | --- | --- | --- |
| **E**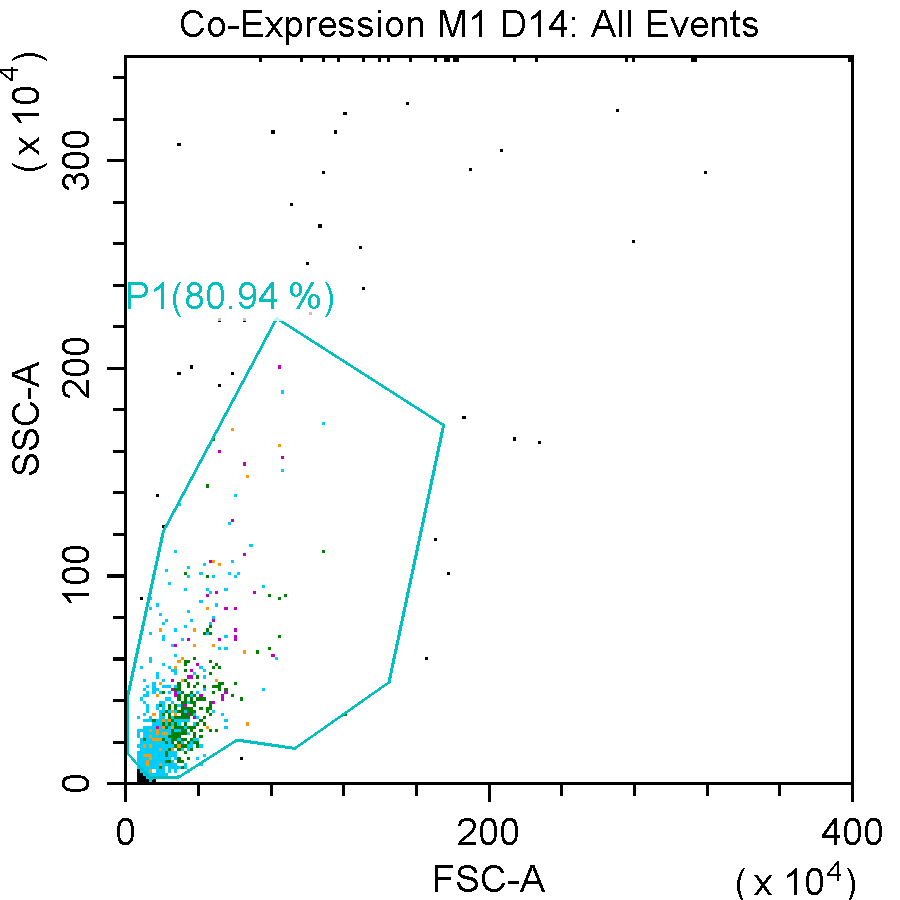 | **F**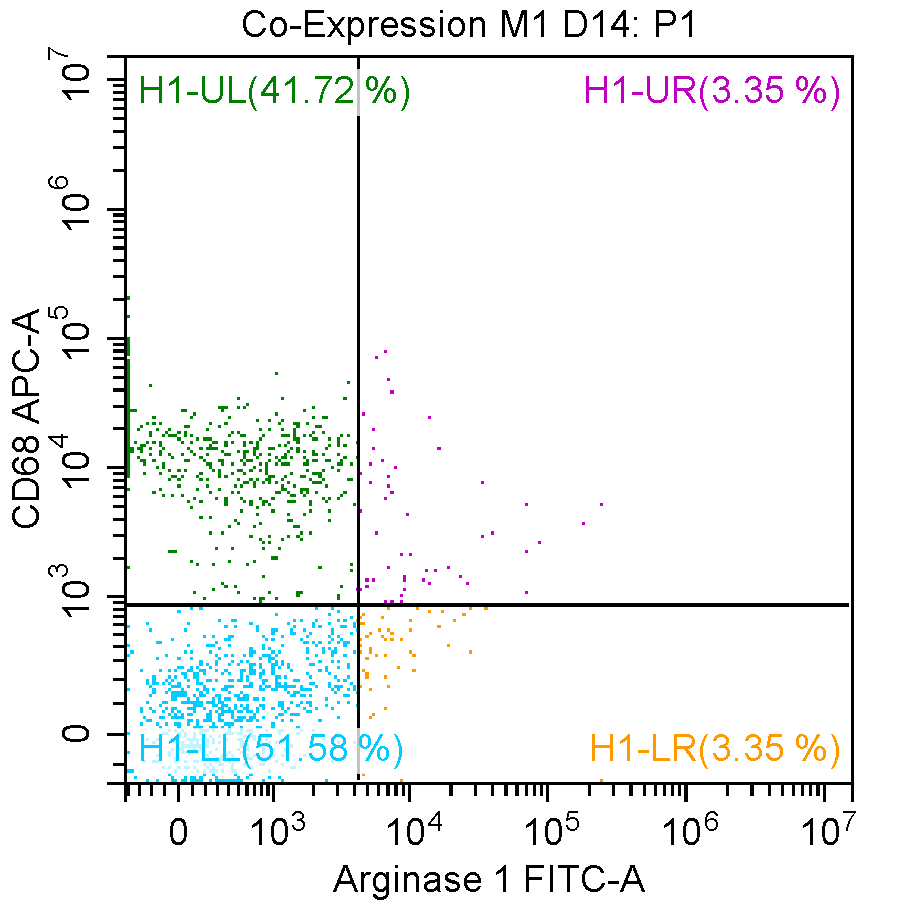 | **G**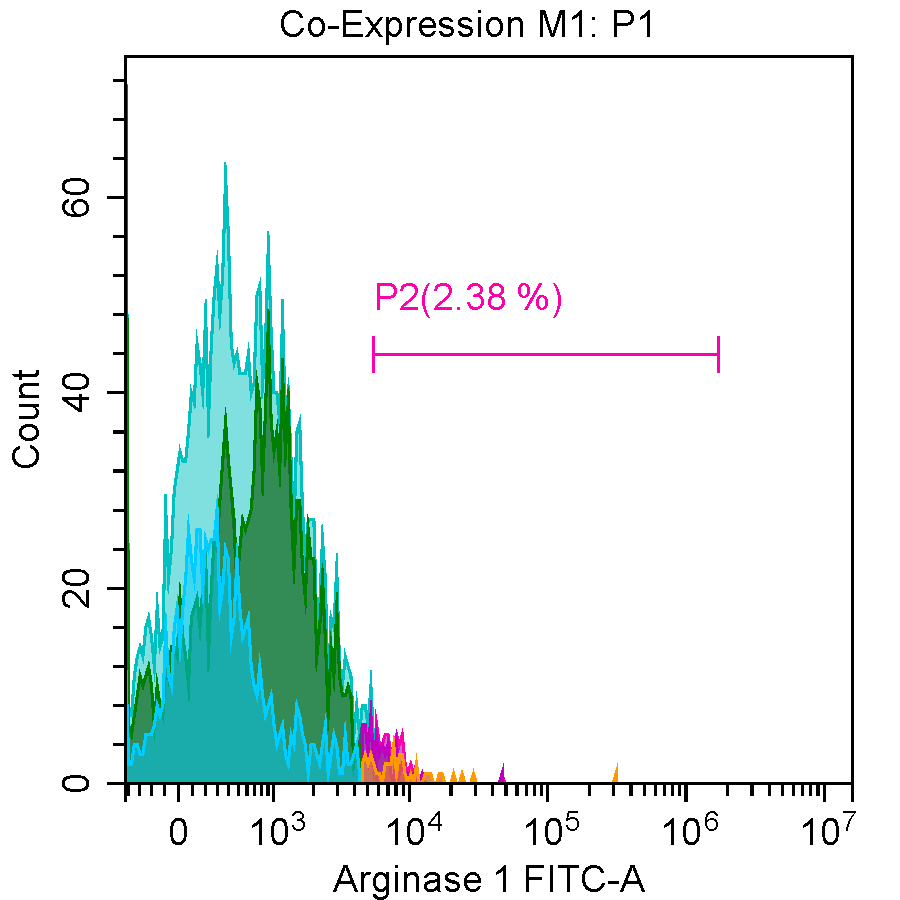 | **H**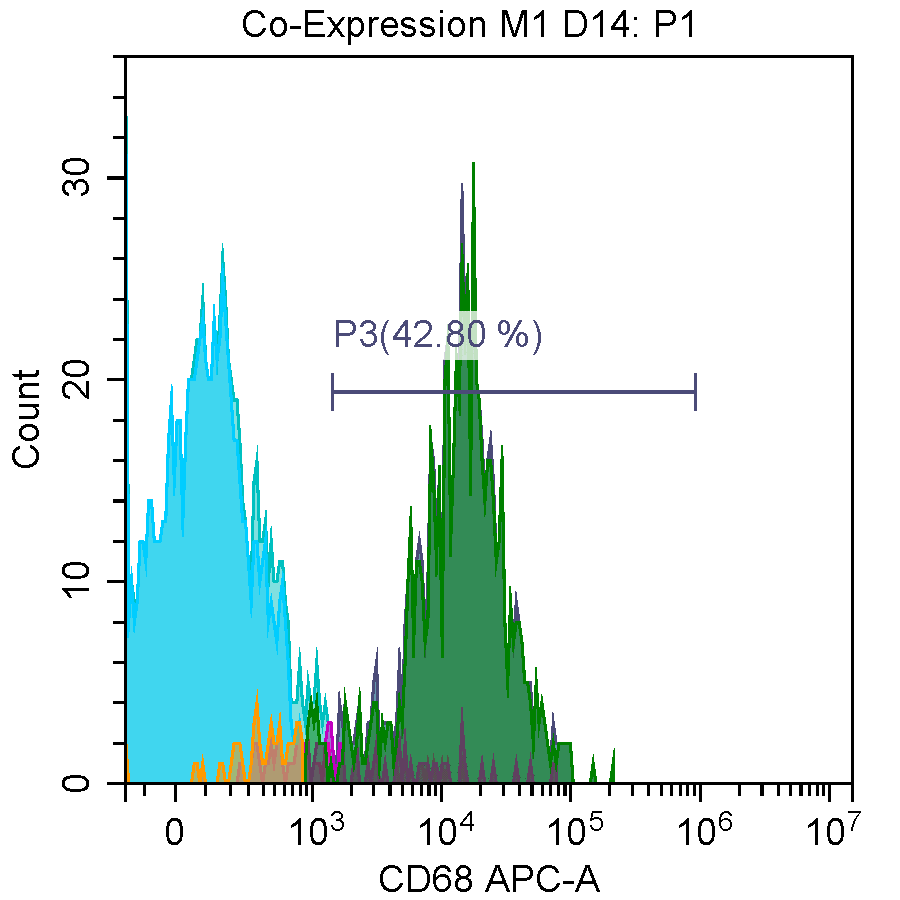 |
| **I**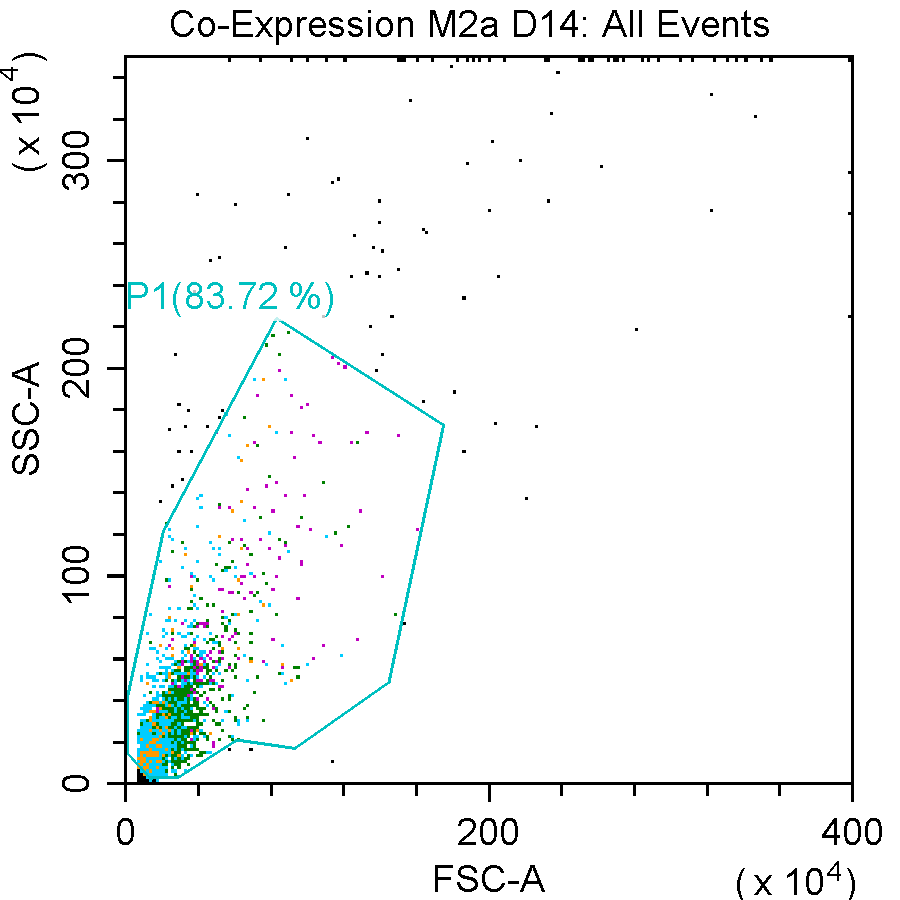 | **J**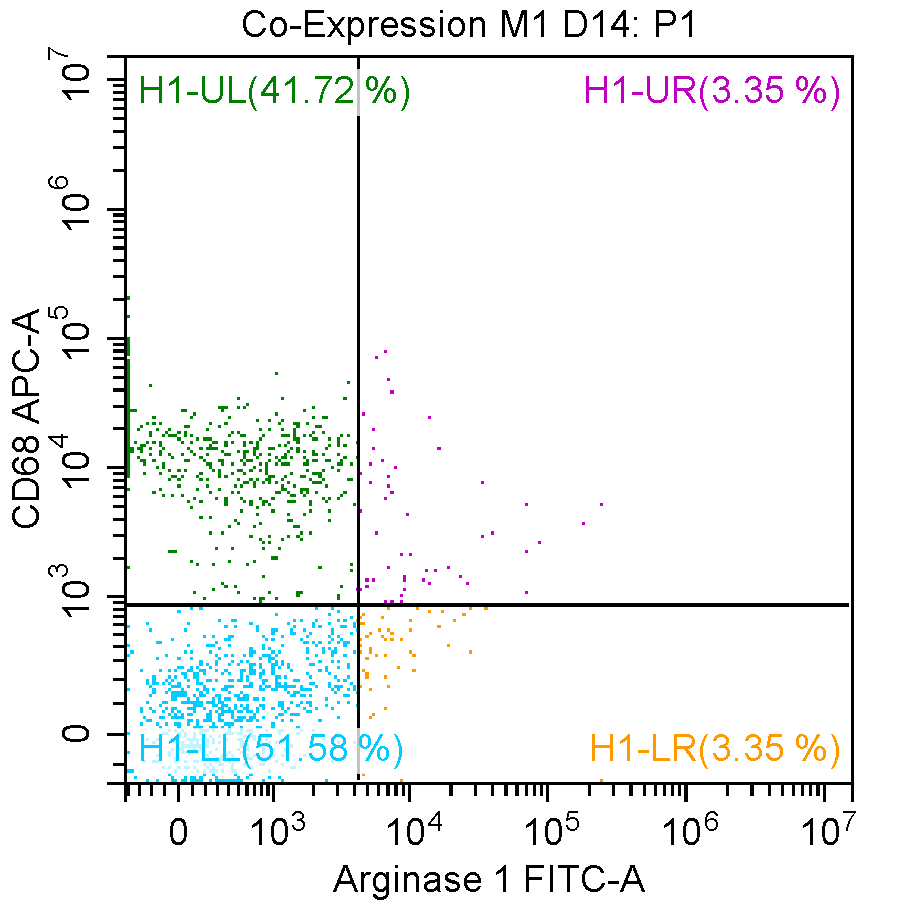 | **K**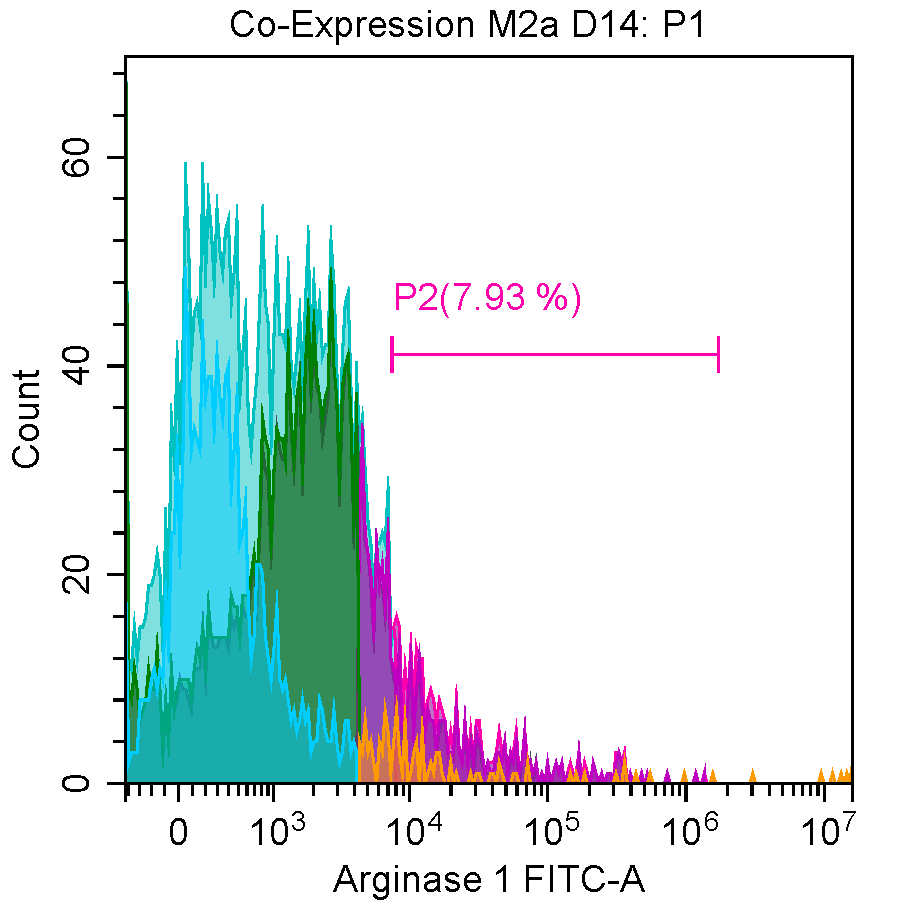 | **L**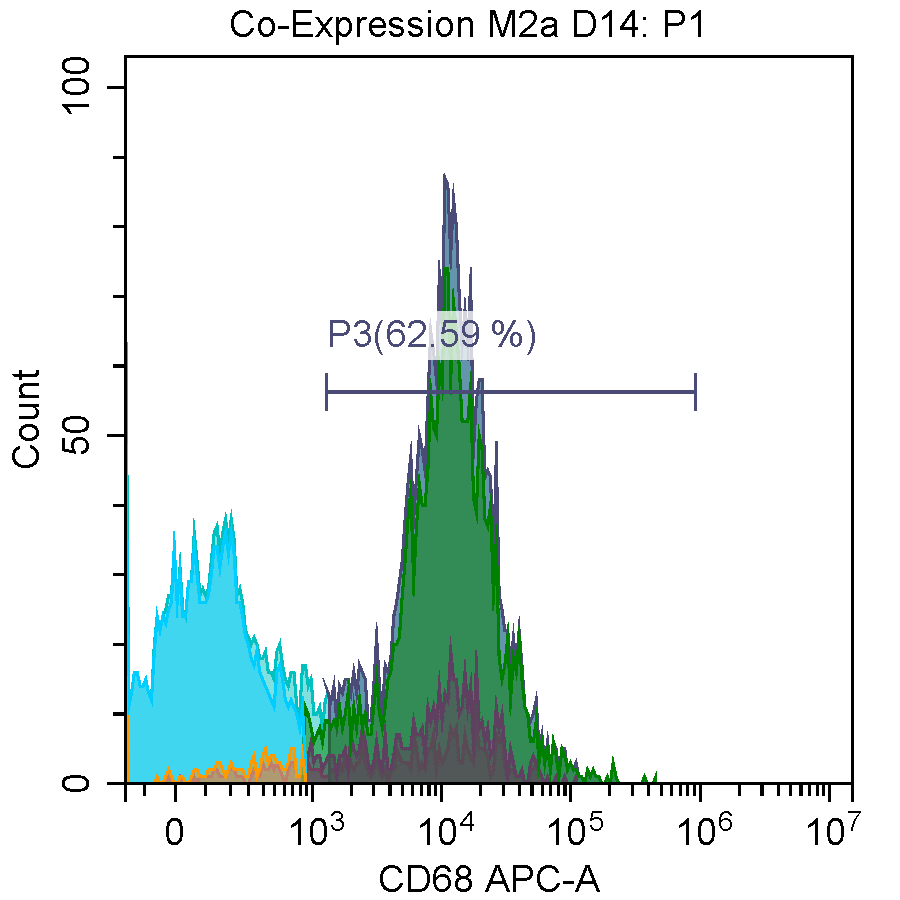 |
| **M-** Flow Cytometry Analysis of CD68 and Arginase -1 in BMDM at Day 14 Polarization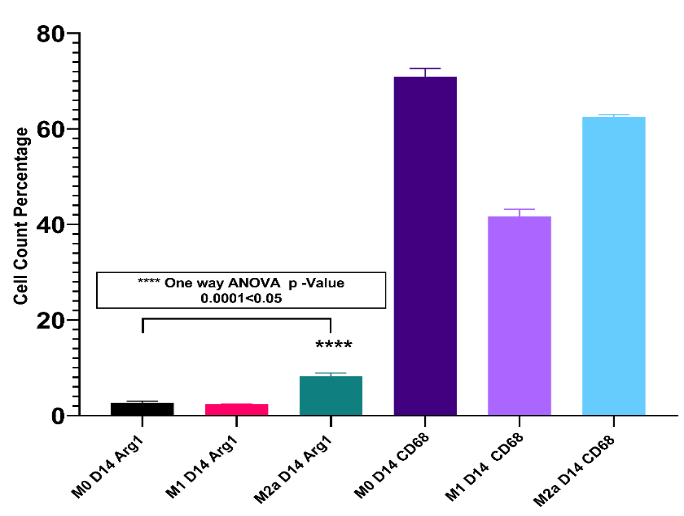 | | | |

**Figure 10S.** **Flow cytometry analysis of phagocytosis markers CD68 and Arginase expression in macrophages at day 14 polarization.**

The figure shows flow cytometry analysis for co-expression of Cd68 and Arginase 1 at M0, M1, and M2a day14 polarization. Samples were gated on 81%, and Cd-68 expression was assessed at APC - Filter, and Arginase-1 was read at FITC- Filter. where (A, E, and I) represent the gating for 5000 events (event= single cell) inside scatter plots (SSC-A) on X-axis and forwarded scatter plots (FSC-A) on Y-axis. (A, E, and I) for M0, M1, and M2a lineages, respectively. B, F, and J are quadrant plots of M0, M1, and M2a, respectively. C, G, and K show fluorescence Peak signals for Arginase -1 expression in M0, M1, and M2a cells. The results show lower expression of arginase-1 at M1 and M2a lineages. D, H, and L show the fluorescence signal peaks for CD68 expression in M0, M1, and M2a, respectively. Figure (M) shows the statistical significance of flow cytometry studies of Arginase 1 and CD-68 expression (n=3, p-value0.0001<0.05). CD-68 showed no significant difference between all samples.

| A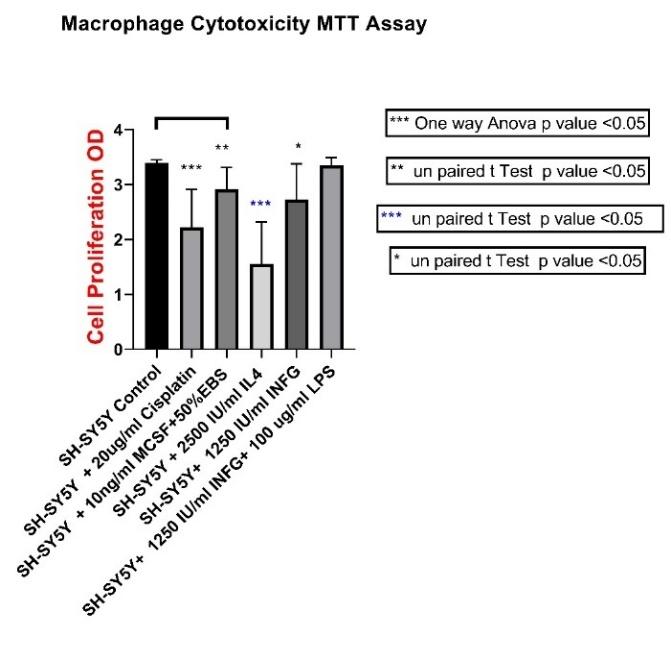 | B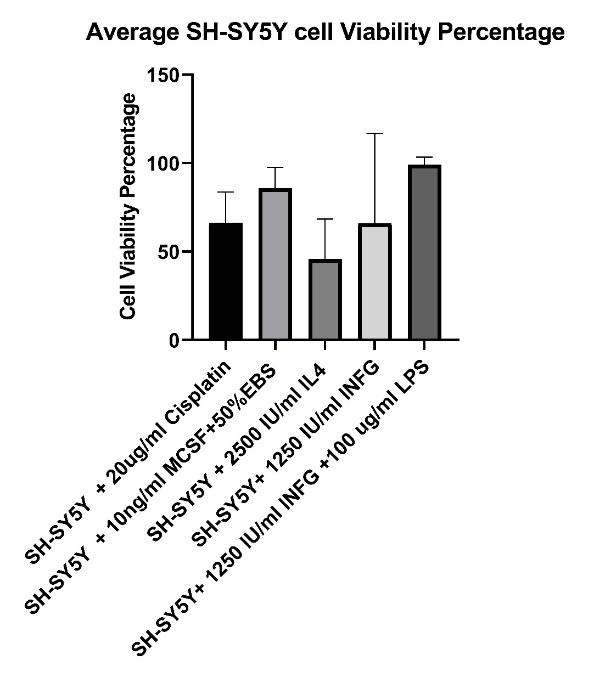 |
| --- | --- |
| C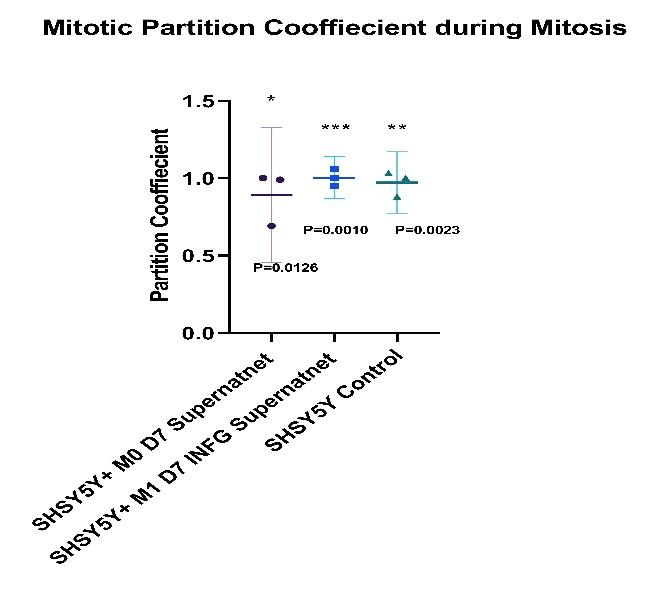 |  |

**Figure 11S. Cytotoxicity assay of Neuroblastoma cell line SHSY5Y.**

Cytotoxicity assay (MTT) of neuroblastoma cell line SHSY5Y cultures in Bone marrow-derived macrophages conditioned media. Cells were seeded in 96-well plates for 48 h to reach confluence before adding the Bone marrow-derived macrophages media. Neuroblastoma co-cultured in 2500 IU IL4 showed the most significant decrease in cell proliferation when compared to the negative control (p-value <0.05). Cells treated with 20 ug/ml Cisplatin also showed decreased proliferation (p-value <0.05). Significant proliferative activity was observed in SHSY5Y incubated in M1 conditioned media compared to control.

| **A**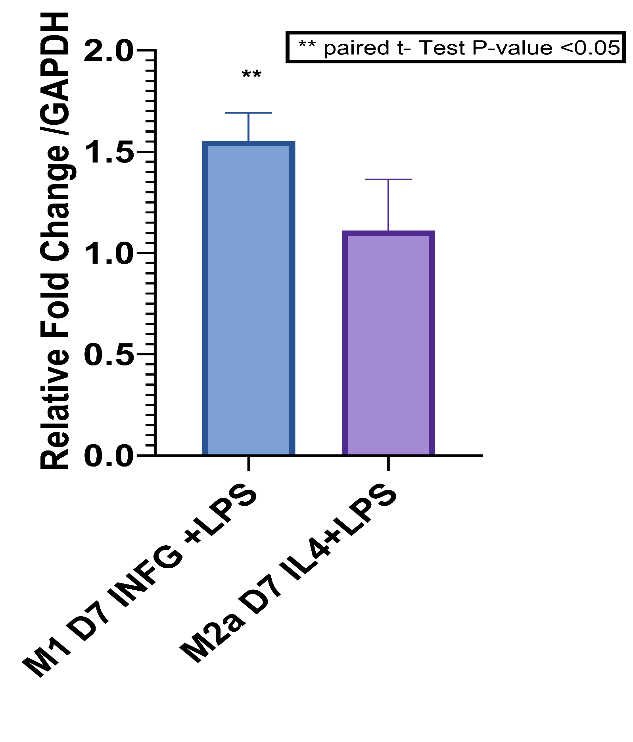 | 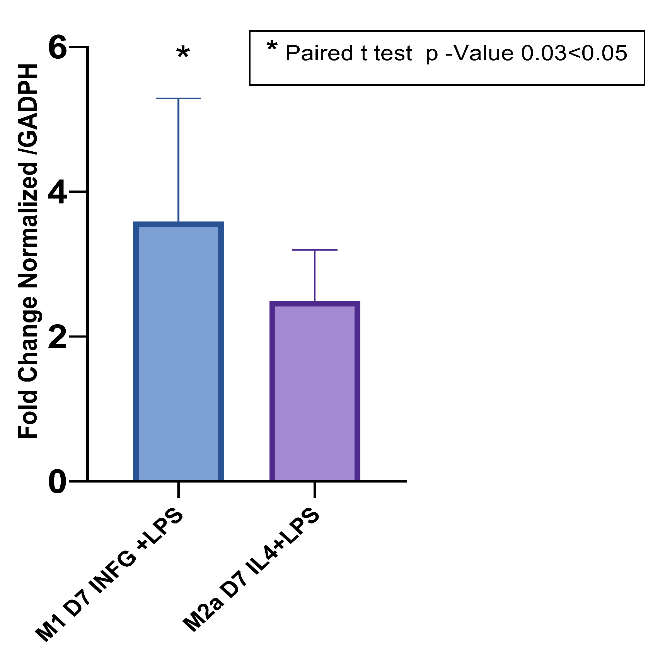  **B** |
| --- | --- |

**Figure 12S.** **Relative fluorescence intensity of LC3A&B.**

A) shows the statistical representation of LC3A fold change in both M 1 and M2a at day 7 polarization normalized to GAPDH as endogenous control and M0 lineage as control, (n=4, p-value- 0.0012< 0.05). Figure (b) Bar plot shows a statistical representation of LC3B fold change in both M 1 and M2a at day 7 polarization normalized to GAPDH as endogenous control and M0 lineage as control. Paired t-test used to calculate significance (n=4, p-value- 0.03 < 0.05).

| **A- Gene Expression Fold Change of Atg16L1-1 at Day7**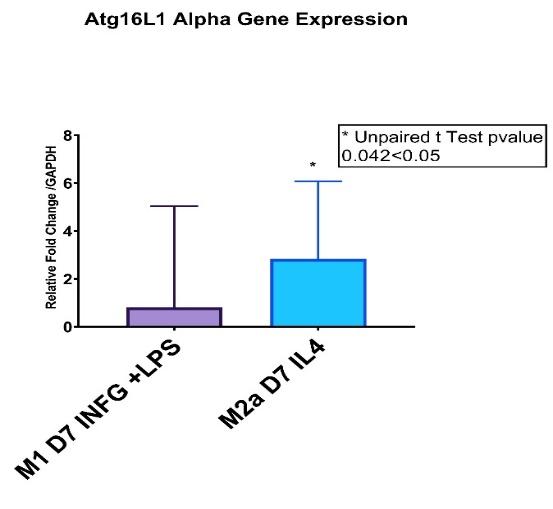 | **B- Gene Expression Fold Change of Atg16L1-1 at Day14**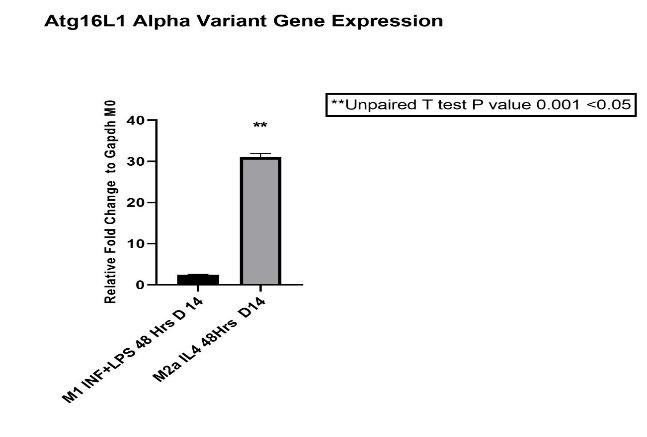 |
| --- | --- |
| **C- Gene Expression fold change of Atg16L1-3 at Day7**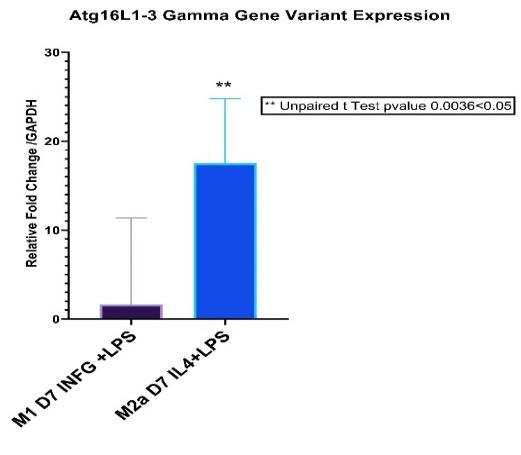 | **D- Gene Expression fold change of Atg16L1-3 at Day14** 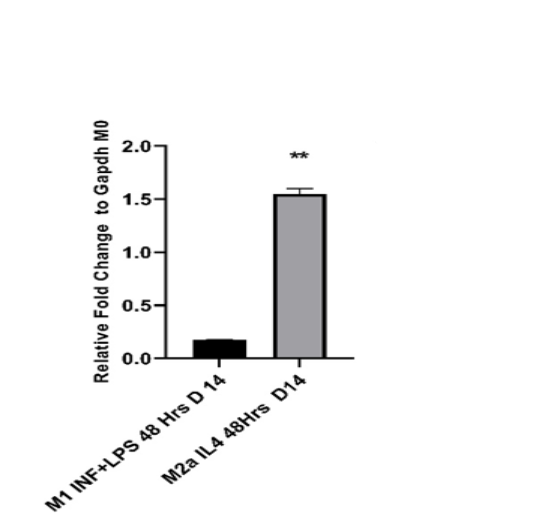 |
| **E- Gene Expression fold change of Vamp7 at Day7** 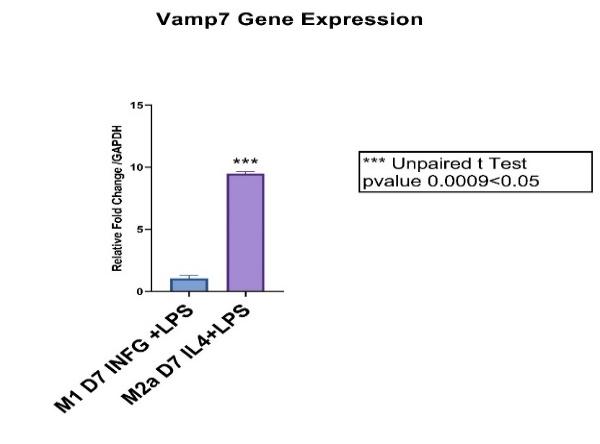 | **F- Gene Expression fold change of Vamp7 at Day14**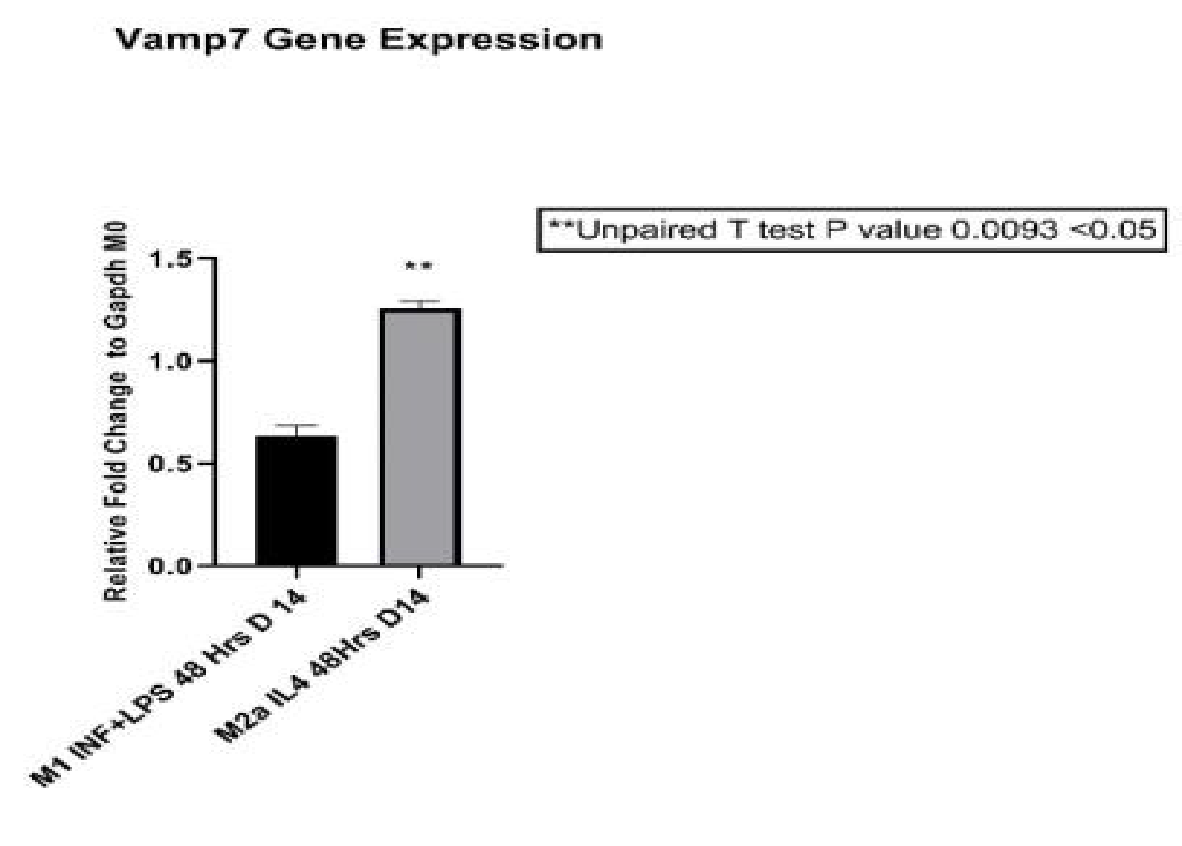 |

**Figure 13S. Atg16L1-1, Atg16L1-3, and Vamp7 Gene Expression.**

Figures show the gene expression of Atg16L1-1, Atg16L1-3, and Vamp7 at days 7 and 14 polarization normalized to GAPDH. A and B are bar plots showing the relative fold change of Atg16L1-1 alpha variant in M1 and M2a at day 7 (A) polarization (n=4, p-value-0.042 < 0.05) and at day 14 polarization (n=4, p-value 0.001 < 0.05). C and D are bar plots showing the relative fold change of Atg16L1-3 Gamma variant in M1 and M2a at day 7 (C) (n=4, p-value-0.0036 < 0.05) and (D) day 14 polarization (n=4, p-value -0.0013 < 0.05). E and F show the relative fold change of the Vamp7 variant in M1 and M2a at day 7 (n=4, p-value-0.0009 < 0.05) and day 14 (n=4, p-value -0.0093< 0.05) polarization. M2a lineage showed the highest Atg16L1-1, Atg16L1-3, and Vamp7 fold change at both 7- and 14-days polarization.

| **A- Flow cytometry analysis of CD-68 expression in Bone marrow macrophages with Bafilomycin-A.**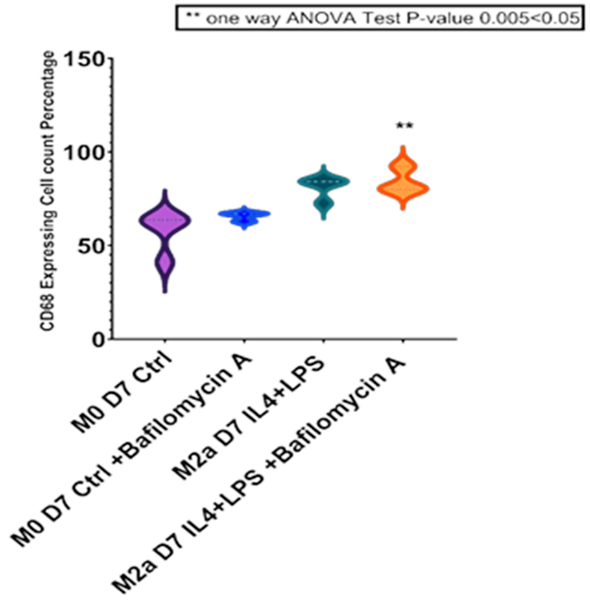 | 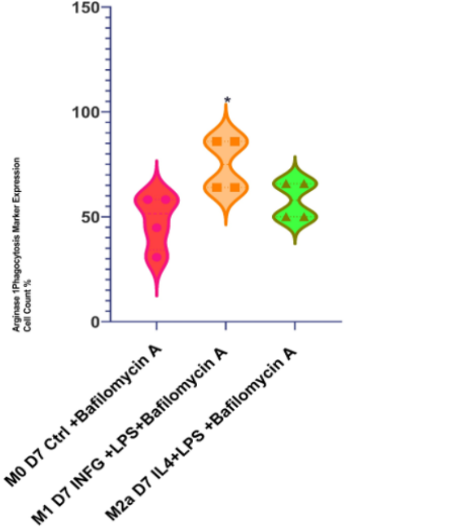  **B- Flow cytometry analysis of Arg-1 expression in Bone marrow macrophages with Bafilomycin-A.** |
| --- | --- |
| **C- CD-68/Arg-1 double-positive bone marrow macrophages**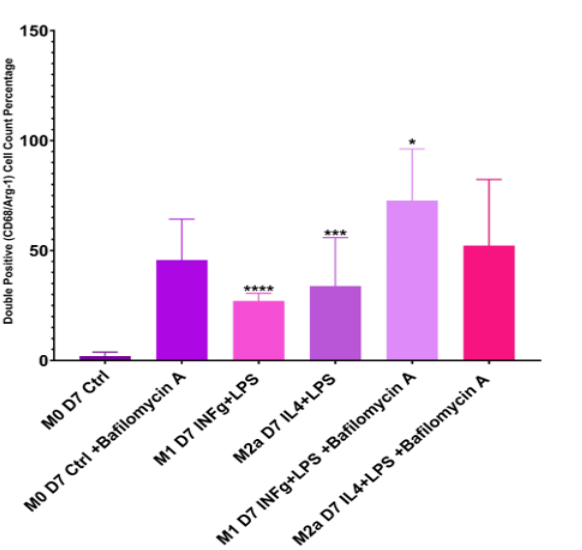 | |

**Figure 14S. Flow cytometry analysis of CD-68 and Arg-1 Expression with Bafilomycin-A**

A) violin plot showing the expression of CD-68 in M0, M2a and M2a incubated with Bafilomycin-A (n=3, p-value 0.0024<0.05). M2a cells + Bafilomycin-A showed the highest expression levels of CD-68. B) violin plot showing the total expression Arg-1 in M0, M1, and M2a. M1 cells showed the highest expression levels of Arg-1 (n=3, p-value 0.02<0.05). C) Bar plot showing double-positive cells expressing both CD-68 and Arg-1 in M0, M1, and M2a at day 7 polarization incubated with Bafilomycin-A. (n=3, p-value <0.0001).

| 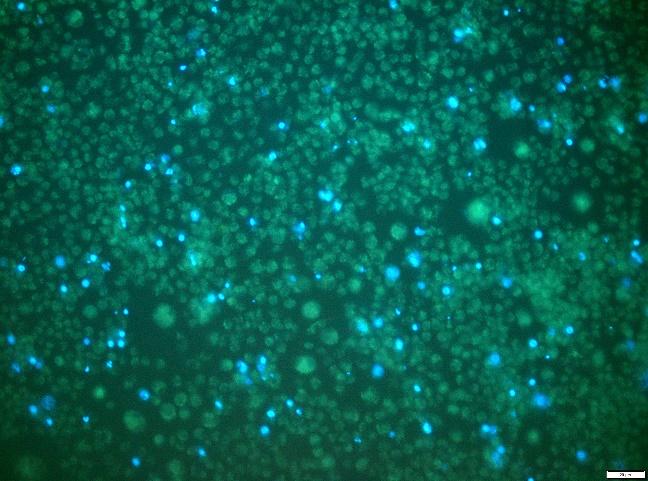 **A. M0 D7 EBS 16 Hrs. + E. coli** | **B. M1 D7 EBS 16 Hrs. + E. coli**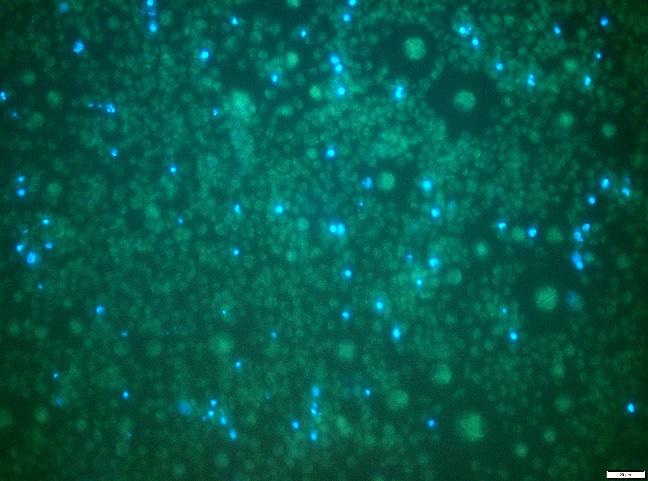 |
| --- | --- |
| 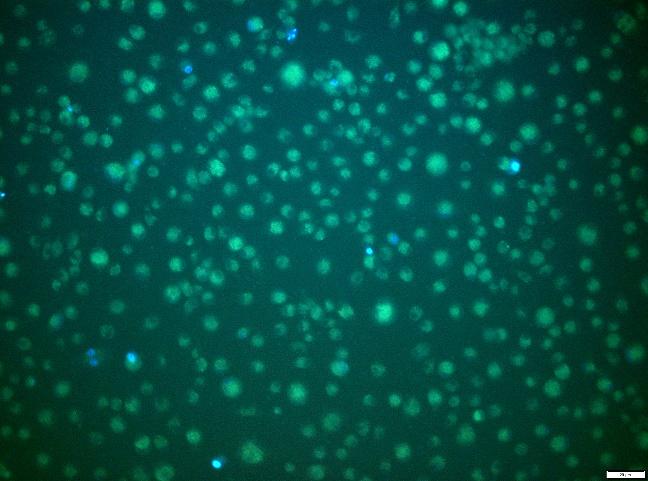  **C. M2a D7 EBS 16 Hrs. + E. coli** | **D. Phagocytosis of E. Coli**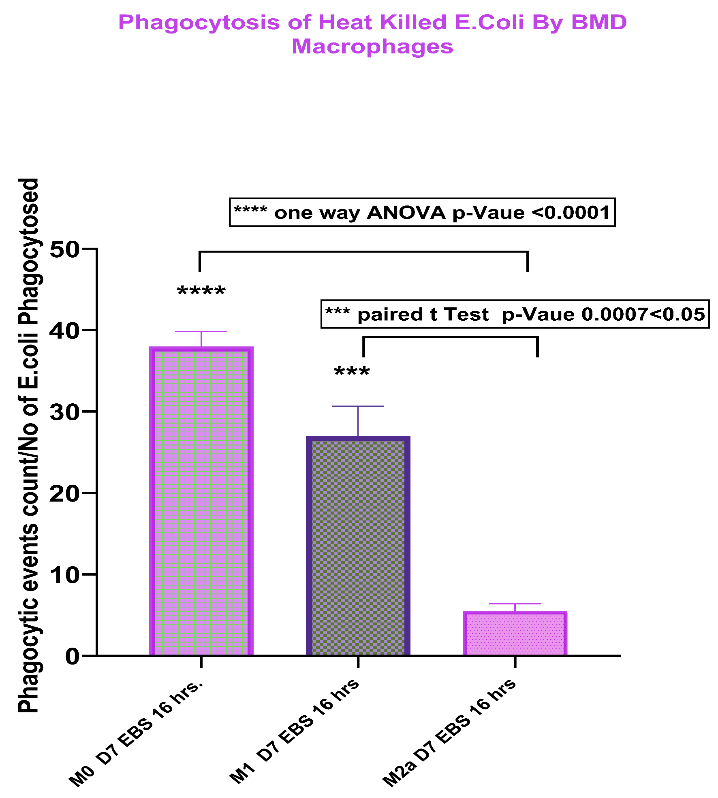 |

**Figure 15S. Phagocytosis Assay.**

Immune staining studies (A, B, and C) using mak38 autophagy detection kit. Cells stained in cyan M0, M1, and M2a at day 7 were incubated in Earle balanced salt for 16 hrs. Heat-killed E. coli (10 µl) stained with DAPI blue stain was added, and phagocytic events were captured using a fluorescence microscope. D) Bar plot showing the statistical significance (n=5 images, p-value <0.0001). Autophagy decreased the phagocytic activity of M2a compared to M1 and M0.
